# Economic Evaluation of Cost and Time Required for a Platform Trial vs Conventional Trials

**DOI:** 10.1001/jamanetworkopen.2022.21140

**Published:** 2022-07-12

**Authors:** Jay J. H. Park, Behnam Sharif, Ofir Harari, Louis Dron, Anna Heath, Maureen Meade, Ryan Zarychanski, Raymond Lee, Gabriel Tremblay, Edward J. Mills, Yannis Jemiai, Cyrus Mehta, J. Kyle Wathen

**Affiliations:** 1 Experimental Medicine, Department of Medicine, University of British Columbia, Vancouver, British Columbia, Canada; 2 Department of Health Research Methods, Evidence, and Impact, McMaster University Health Sciences Centre, Hamilton, Ontario, Canada; 3 Cytel, Inc, Waltham, Massachusetts; 4 Child Health Evaluative Sciences, The Hospital for Sick Children, Toronto, Ontario, Canada; 5 Dalla Lana School of Public Health, University of Toronto, Toronto, Ontario, Canada; 6 Department of Statistical Science, University College London, London, United Kingdom; 7 Interdepartmental Division of Critical Care, Hamilton Health Sciences, Critical Care, Hamilton, Ontario, Canada; 8 Department of Internal Medicine, Section of Critical Care, University of Manitoba, Winnipeg, Manitoba, Canada; 9 Department of Internal Medicine, Section of Hematology/Medical Oncology, University of Manitoba, Winnipeg, Manitoba, Canada; 10 Department of Biostatistics, Harvard T.H. Chan School of Public Health, Cambridge, Massachusetts

## Abstract

**Importance:**

Platform trial design allows the introduction of new interventions after the trial is initiated and offers efficiencies to clinical research. However, limited guidance exists on the economic resources required to establish and maintain platform trials.

**Objective:**

To compare cost (US dollars) and time requirements of conducting a platform trial vs a series of conventional (nonplatform) trials using a real-life example.

**Design, Setting, and Participants:**

For this economic evaluation, an online survey was administered to a group of international experts (146 participants) with publication records of platform trials to elicit their opinions on cost and time to set up and conduct platform, multigroup, and 2-group trials. Using the reported entry dates of 10 interventions into Systemic Therapy in Advancing Metastatic Prostate Cancer: Evaluation of Drug Efficacy, the longest ongoing platform trial, 3 scenarios were designed involving a single platform trial (scenario 1), 1 multigroup followed by 5 2-group trials (scenario 2), and a series of 10 2-group trials (scenario 3). All scenarios started with 5 interventions, then 5 more interventions were either added to the platform or evaluated independently. Simulations with the survey results as inputs were used to compare the platform vs conventional trial designs. Data were analyzed from July to September 2021.

**Exposure:**

Platform trial design.

**Main Outcomes and Measures:**

Total trial setup and conduct cost and cumulative duration.

**Results:**

Although setup time and cost requirements of a single trial were highest for the platform trial, cumulative requirements of setting up a series of multiple trials in scenarios 2 and 3 were larger. Compared with the platform trial, there was a median (IQR) increase of 216.7% (202.2%-242.5%) in cumulative setup costs for scenario 2 and 391.1% (365.3%-437.9%) for scenario 3. In terms of total cost, there was a median (IQR) increase of 17.4% (12.1%-22.5%) for scenario 2 and 57.5% (43.1%-69.9%) for scenario 3. There was a median (IQR) increase in cumulative trial duration of 171.1% (158.3%-184.3%) for scenario 2 and 311.9% (282.0%-349.1%) for scenario 3. Cost and time reductions in the platform trial were observed in both the initial and subsequently evaluated interventions.

**Conclusions and Relevance:**

Although setting up platform trials can take longer and be costly, the findings of this study suggest that having a single infrastructure can improve efficiencies with respect to costs and efforts.

## Key Points

**Question:**

What are the cost and time requirements of conducting a single platform trial vs conventional clinical trials?

**Findings:**

In this economic evaluation, according to the opinions of platform trial experts elicited and scenarios constructed from the longest ongoing platform trial, conducting a series of 2-group trials for evaluation of 10 interventions showed median (IQR) increases in total trial costs by 57.5% (43.1%-69.9%) and cumulative trial duration by 311.9% (282.0%-349.1%) compared with conducting a single platform trial.

**Meaning:**

These findings suggest that despite having larger initial setup requirements, consolidating clinical evaluation of multiple interventions into a single platform trial can drastically reduce cost and efforts.

## Teaser

This economic evaluation compares the cost and time associated with platform trials vs conventional trials.

## Introduction

Conventionally, clinical trials have a defined end and only compare prespecified intervention(s), meaning that therapeutic discoveries made during the trial are evaluated in a new trial.^1,2,3^ This results in multiple independent trials, requiring new infrastructure for each shorter-term evaluation and more patients randomized to placebo or standard-of-care compared with platform trials.^4,5^

Platform trials are randomized clinical trials that allow for multiple interventions to be simultaneously compared and new interventions to be added after the trial is initiated.^1,6,7,8^ They typically aim to continue for an extended period or in perpetuity with interventions entering and leaving the platform at different times.^1,2^ Many published reviews on platform trials describe the statistical efficiencies of platform trials over conventional trial approaches.^6,7,9,10,11,12^ There is little guidance, however, on substantive resources to establish and maintain platform trials.

Because of the scale and perpetual nature of platform trials, their setup can be prohibitively complex in terms of organization, time, and costs, more so than conventional trials.^13,14,15,16^ We undertook, therefore, an economic evaluation of platform trials, based on Systemic Therapy in Advancing or Metastatic Prostate Cancer: Evaluation of Drug Efficacy (STAMPEDE), the first platform trial to be conducted.^6^ Launched in 2005, the trial has included 10 interventions for advanced prostate cancer in the UK over 17 years, with complete evaluations of 8 systematic therapies and 2 currently included in the trial.^17,18^

## Methods

The study followed the Consolidated Health Economic Evaluation Reporting Standards (CHEERS) reporting guideline for economic evaluations. The study comprised a simulation model that used secondary literature and an anonymous online survey to inform model inputs. As no data from actual patients were collected or evaluated, institutional review board approval and informed consent were not required, in accordance with the Tri-Council Policy Statement 2, Article 2.2.^19^

### Study Overview

We administered an online survey to elicit expert opinions on time and cost requirements of platform, conventional 2-group, and multigroup trials. We then designed 3 scenarios that compared a platform trial with conventional trials using STAMPEDE as our real-life example since it is not possible to determine the number of interventions and when they will be added into platform trials. For each scenario, we performed trial simulations to estimate the sample size and follow-up duration required to evaluate 10 interventions, as currently in the case of STAMPEDE; then, according to these simulation outputs, we calculated the setup and total cost and time (person-years) using the elicited opinions.

### Survey of Platform Trial Experts

We surveyed a group of experts determined according to the publication record of platform trials using purposive sampling. We had a comprehensive list of peer-reviewed publications, conference abstracts, and trial registry records that discussed and/or reported on platform trials from a previous landscape analysis of master protocols (90 records).^6^ We reviewed the individual records to extract an email list of first, last, and corresponding authors for our survey.

The survey was administered using SurveySparrow.com in April 2021 (eMethods 1 in the Supplement). The survey included 5 questions about the respondent and 15 questions related to trial setup (7 questions), conduct (6 questions), and analyses (2 questions). These questions stemmed from an article by Moore et al^20^ that discussed estimated costs required for clinical trials. Trial setup questions asked for time and cost requirements of developing a trial protocol, obtaining study approval, managing a trial database, and setting up trial sites. Trial conduct questions asked for monthly costs required to recruit and follow up patients and manage clinical trial sites and databases, cost required to conduct statistical analyses, and, for platform trials, time and cost required to add a new intervention to the platform were also asked.

### Economic Evaluation Through Simulations

**Figure 1.**
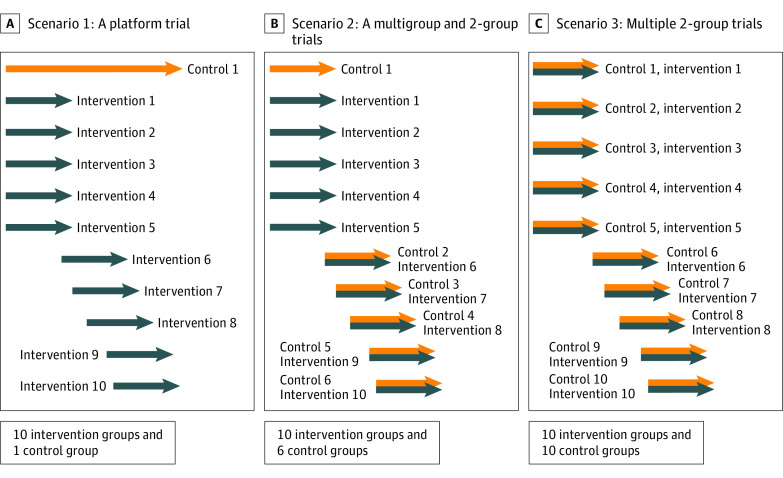
Competing Scenarios: A Platform Trial vs Multiple Conventional Clinical Trials According to Systemic Therapy in Advancing or Metastatic Prostate Cancer: Evaluation of Drug Efficacy (STAMPEDE), 3 competing scenarios that would all evaluate 10 interventions are illustrated here. The first scenario involves a single platform trial with a single common control group that would be used for evaluation of 10 intervention groups. The second scenario involves a 6-group trial followed by 5 independent 2-group trials (10 intervention plus 6 control groups), and the third scenario involves 10 independent 2-group trials (10 intervention plus 10 control groups). The same start time for all 3 competing scenarios were used in our simulations.

We compared the setup and total cost and time required to evaluate 10 interventions in 3 trial design scenarios (Figure 1). Scenario 1 is a platform trial that begins with 5 interventions and a common control group with 5 additional interventions that would be added to the platform thereafter, as in the case of STAMPEDE.^21,22^ Scenario 2 begins like scenario 1 but new emerging interventions would be independently evaluated in 2-group trials (1 6-group trial plus 5 2-group trials). Finally, scenario 3 is 10 independent 2-group trials. We used the entry dates into STAMPEDE as the trial initiation dates in all scenarios, thus allowing the trials to run concurrently in our evaluation.

### Trial Simulation Assumptions

We used trial simulations to estimate the sample size and trial duration of each scenario (eTable 1 in the Supplement). Similar to STAMPEDE, we simulated event-driven trials in which the interim and final analyses used a log rank test that would be triggered according to the number of events observed in the concurrent control group (patients randomized to control during the same time as the intervention group ).^21,22^ Equally in all scenarios, the evaluation of each intervention could undergo 3 interim futility analyses according to failure-free survival (FFS) when 114, 215, and 334 FFS events are observed in the concurrent control group with statistical thresholds of FFS hazard ratios (HRs) of 1.0, 0.92, and 0.89 for futility; if necessary, final analysis was conducted according to overall survival (OS), when 400 deaths are observed in the control group.^21,22^ When an intervention is dropped early, randomization to that group is discontinued, so the overall sample size requirement is reduced.

Since operating characteristics will vary between different treatment outcomes, we conducted 3 scenario analyses to consider their outcomes. At the time the simulations were conducted (September 2021), STAMPEDE had published their findings on the first 7 interventions.^23,24,25,26^ For these interventions, we used the reported point estimate HRs on FFS and OS as the base case, the lower CI as the best case, and the upper CI as the pessimistic case. For the subsequent 3 interventions without published results, we used the original target outcomes of an HR of 0.75 for FFS and OS.^21,22^ The best-case for these groups assumed an HR of 0.5625 (twice the target outcomes), and the pessimistic case assumed that these interventions would have no changes (HR = 1.00).

We assumed a maximum recruitment of 443 patients for each intervention group. As in the case of STAMPEDE, we specified an unequal allocation in favor of the control group for the first 5 experimental interventions (2:1:1:1:1:1), allowing the control group to be twice as large in scenarios 1 and 2.^21,22^ The recruitment to the control was reduced to 443 for the evaluation of subsequent interventions to match the equal allocation ratio that was later adopted in STAMPEDE. As reported, we assumed that a platform trial would have 120 sites that would recruit 500 patients per year.^21,22^ Assuming an equal enrollment rate per site, we specified that there would be a total of 80 sites for multigroup trials and 50 sites for 2-group trials based on our collective experience running clinical trials and, more recently, platform trials. Platform trials usually have a larger recruitment target, so they can involve a larger number of sites. Other requirements of trial setup and adding new interventions (for platform trials only) estimated from the expert surveys were used for trial simulations.

### Cost Simulations and Analyses

According to the trial assumptions mentioned already, simulations were performed 5000 times (eFigure 1 in the Supplement). In addition to duration and sample sizes estimated from trial simulations, we used the top-down costing method^27^ in which the trial setup, conduct and analysis^28^ were estimated separately using the parameters from the expert survey (eTable 1 in the Supplement). The costing was conducted from the budgetary perspective of the trial funder. The variation in the cost of the trials was assumed to follow a lognormal distribution and a normal distribution for time. Method of moments were used to estimate the SD for each parameter (eMethods 2 in the Supplement).^29^ All costs are reported in 2021 US dollars. No discounting for costs was considered for this study as budgets are determined for each year in nominal currency.

### Statistical Analysis

Descriptive statistics of the estimated cost and time requirements are presented. We calculate the relative differences of scenarios 2 and 3 with respect to the platform trial scenario by taking the difference between each simulation iteration matched across all simulations to obtain a sample of the distribution of relative differences. We present the median and IQR for these differences. Simulations described already used an open-source R package called Optimize Clinical Trials On Platforms Using Simulation Update (OPTOPUS).^30^ The cost simulations used R version 4.0.3, and figures were produced using the ggplot2 package, version 3.3.5 (R Project for Statistical Computing). Documentation and source codes for this study are available on GitHub.^31^ Data were analyzed from July to September 2021.

## Results

### Survey Results

We identified 146 email contacts of experts according to their publication record of platform trials. A total of 16 experts (11.0%) completed the survey. Most respondents were residents of the US (6 respondents) or Canada (5 respondents) and indicated their current employment in the private sector (11 respondents), with 1 respondent employed in both private and public sectors. Most respondents indicated having clinical trial experience in oncology therapeutics (11 respondents).

The Table summarizes expert opinions on cost and time requirements to set up and conduct platform, multigroup, and 2-group trials. The setup requirements were generally higher for a platform trial than the conventional trials. For instance, the estimated mean (SD) cost for master protocol development for a platform trial was $155 667 ($34 347). The estimated mean (SD) costs of protocol developments were lower for multigroup trials ($136 667 [$22 480]) and 2-group trials($123 333 [$23 245]). The mean (SD) estimated times required to develop the protocol for multigroup (5.09 [2.26] months) and 2-group trials (3.92 [1.98] months) were also faster than the platform trial (8.78 [3.83] months). Nevertheless, the mean (SD) estimated cost and time of adding a new intervention into the platform were smaller ($75 626 [$43 528]) and faster (3.00 [1.73] months) than starting a new trial.

**Table.** Survey Results

Parameter	Mean (SD)			
	2-group trial	Multigroup trial	Platform trial	Overall
Setup cost requirements, $US 2021				
Trial protocol development	123 333 (23 245)	136 667 (22 480)	155 667 (34 347)	NA
Trial approvals	151 183 (28 126)	165 367 (27 200)	172 250 (38 538)	NA
Database development	32 500 (30 406)	36 667 (34 763)	42 500 (30 625)	NA
Site setup (per site)^a^	NA	NA	NA	9440 (14 086)
Setup time requirements, mo				
Trial protocol development	3.92 (1.98)	5.09 (2.26)	8.78 (3.83)	NA
Trial approvals	3.67 (2.06)	4.00 (2.40)	6.50 (4.14)	NA
Database development	2.80 (1.30)	3.20 (1.30)	5.40 (1.95)	NA
Trial conduct cost requirements, $US 2021^a^				
Recruitment (per patient)	NA	NA	NA	1300 (476)
Monthly follow-up cost per patient	NA	NA	NA	313 (132)
Monthly site management per site	NA	NA	NA	5000 (3162)
Monthly database management cost	NA	NA	NA	2500 (1061)
Trial analysis cost, $US 2021^a^				
An interim analysis (per group)	NA	NA	NA	12 883 (29 417)
A final analysis (per group)	NA	NA	NA	42 750 (37 053)
Cost required to add a new group, $US 2021^b^	NA	NA	75 626 (43 528)	NA
Time required to add a new group, mo^b^	NA	NA	3.00 (1.73)	NA

Abbreviation: NA, not applicable.

^a^ Costs related to site setup and management, patient recruitment and follow-up, database management, and conduct analyses were assumed to be constant between different types of clinical trials considered in this study.

^b^ Cost and time requirements to add a new group only applied to platform trials, as conventional 2-group and multigroup trials do not allow for new intervention groups to be added.

### Comparison of Simulated Setup Requirements

**Figure 2.**
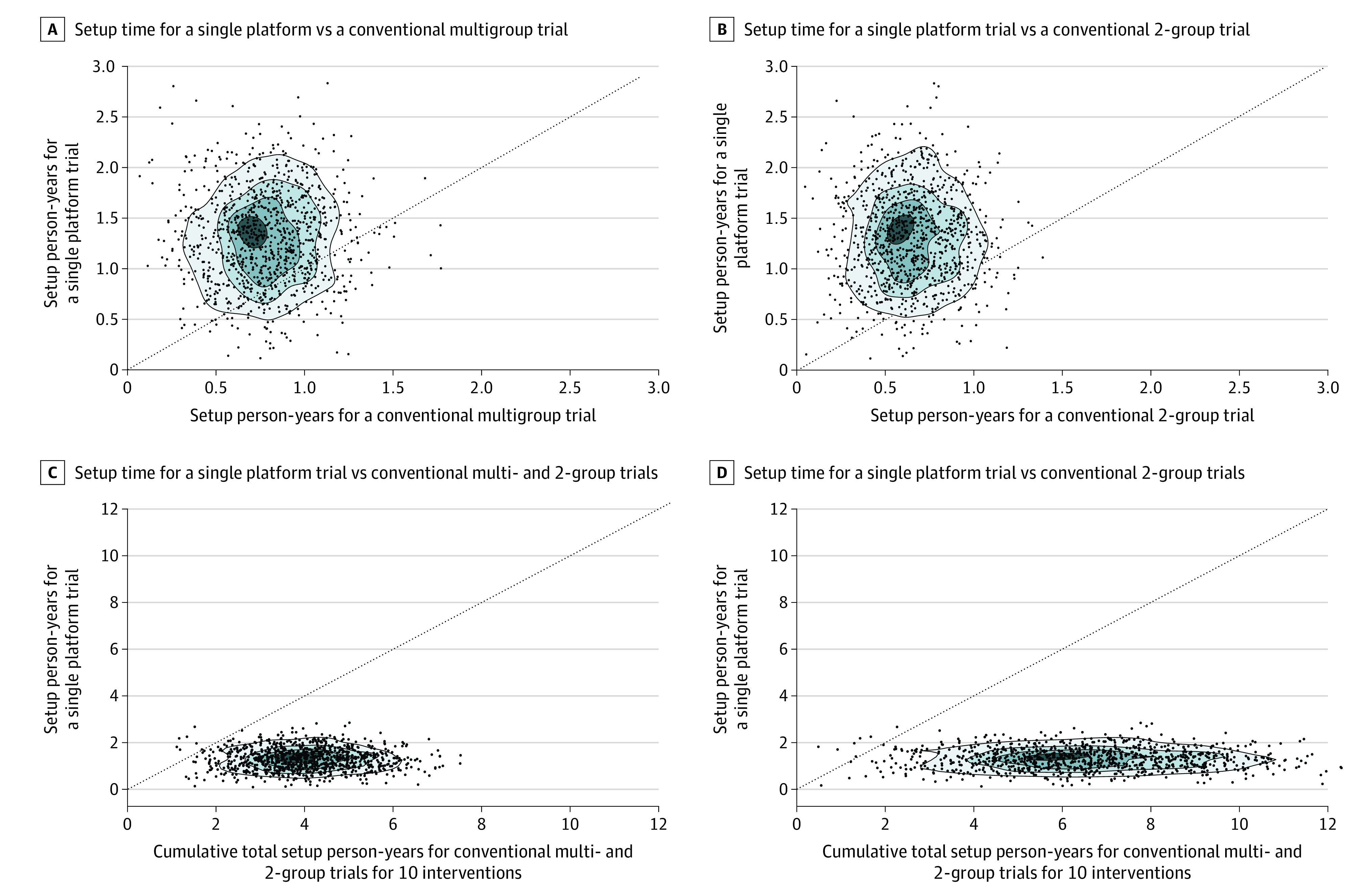
Scatterplots of Setup Times for a Single Platform Trial vs a Multigroup Trial, 2-Group Trial, a Multigroup Plus 2-Group Trials, and 2-Group Trials In panels A and B, scatterplots of setup times for a single platform trial (y-axes) and a single multigroup trial and a single 2-group trial (x-axes) are shown, respectively. Comparison of total setup times between the scenario 1 (a single platform trial) vs scenario 2 that involves 1 multigroup trial plus 5 two-group trials is shown in panel C, and panel D shows the comparison of total setup times between scenario 1 vs scenario 3 (10 two-group trials). Dashed lines denote the lines of equality.

Comparisons of setup time for a single platform trial vs a multigroup trial and a 2-group trial are shown in Figure 2A and Figure 2B, respectively. The mean (SD) simulated setup time for a platform trial was 1.31 (0.45) years. The mean (SD) simulated setup time for a platform trial (1.31 [0.45] years) was longer than a multigroup trial (0.78 [0.25] years) and a 2-group trial (0.65 [0.22] years) (eTable 2 in the Supplement). The mean (SD) simulated setup cost was also higher for a platform trial ($2.24 million [$1.22 million]) than a multigroup trial ($1.59 million [$0.82 million]) and a 2-group trial ($1.09 million [$0.51 million]).

Although the setup requirement of a single trial was the highest for a platform trial, setting up multiple trials to evaluate a total of 10 medical interventions resulted in considerably higher total cumulative setup time (Figure 2C) and costs (Figure 2D) than a platform trial. For instance, when compared with the platform trial, there was a median (IQR) increase of 208.5% (133.1%-330.7%) in the cumulative setup time for scenario 2 that started with a multigroup trial. There was a median (IQR) increase of 398.6% (259.3%-601.5%) for scenario 3 with 10 independent 2-group trials (eTable 2 and eFigure 2 in the Supplement). The total setup costs were also higher for scenarios 2 and 3 than the platform trial (eFigure 3 in the Supplement). Scenario 2 saw a median (IQR) increase of 216.7% (202.2%-242.9%) in setup costs compared with platform design, and the median (IQR) increase was 391.1% (365.3%-437.9%) for scenario 3.

### Comparison of Simulated Total Trial Cost and Time

**Figure 3.**
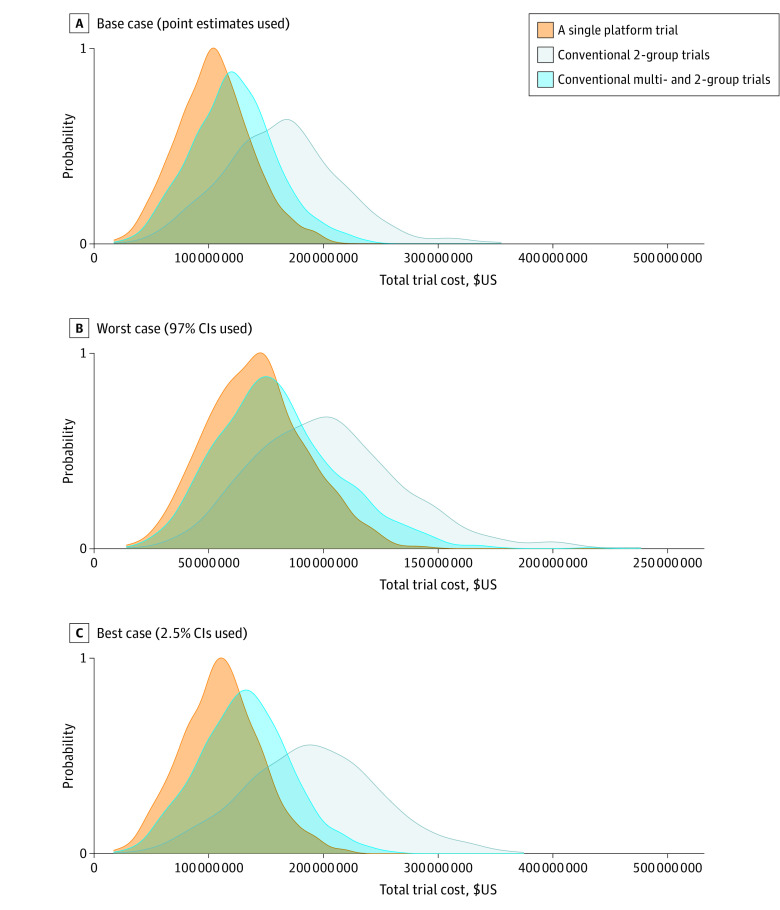
Total Costs of Evaluation of all 10 Interventions: A Platform Trial vs Conventional Multiple Trials For interventions with reported treatment effects (hazard ratio [HR] and 95% CIs) on failure-free survival (FFS) and overall survival (OS) from Systemic Therapy in Advancing or Metastatic Prostate Cancer: Evaluation of Drug Efficacy (STAMPEDE), we used the point estimate as the base case, the lower CI as the best case, and the upper CI as the pessimistic case for our simulations. For the 3 intervention groups without reported results, we assumed the base case FFS and OS to have an HR of 0.75, which was the target treatment effect by the STAMPEDE investigators.^27,28^ The best case for these intervention groups assumed a treatment effect of 0.5625, twice the treatment effect of the target effect, for both FFS and OS, and the pessimistic case assumed that these interventions would have no treatment effects on either outcome (HR = 1.00).

The total costs required to evaluate 10 interventions for platform trial and conventional trial scenarios are shown in Figure 3. In the base case that was based on the point estimate HRs, the estimated mean (SD) trial cost required to evaluate all 10 interventions was $104.95 million ($32.51 million) for the platform trial (eTable 3 and eFigure 4 in the Supplement). Compared with the platform trial, there was a median (IQR) increase of 17.4% (12.1%-22.5%) in total cost for scenario 2, and a median (IQR) increase of 57.5% (43.1%-69.9%) for scenario 3. The magnitude of the increases in cumulative total duration for conventional trial scenarios were considerably higher than the platform trial that had an estimated mean (SD) total duration of 20.75 (1.16) years. The median (IQR) increase in cumulative trial duration was 171.1% (158.3%-184.3%) for scenario 2 and 311.9% (282.0%-349.1%) for scenario 3.

When the cost and time were broken down to the initial interventions (interventions 1-5) that were available at the beginning vs subsequent interventions (interventions 6-10) that could be added to the platform or be evaluated independently, we still observed higher cost and duration in conventional trial scenarios. For the evaluation of the initial interventions, scenario 2 had a smaller magnitude of relative differences against the platform trial in both cost and time than scenario 3. The median (IQR) increase in trial cost for the scenario 2 was 28.0% (5.5%-50.1%) and 158.4% (136.9%-184.1%) for scenario 3. The median (IQR) increase in trial duration was also smaller for scenario 2 (36.1% [30.8%-41.5%]) than scenario 3 (526.3% [429.4%-657.9%])

**Figure 4.**
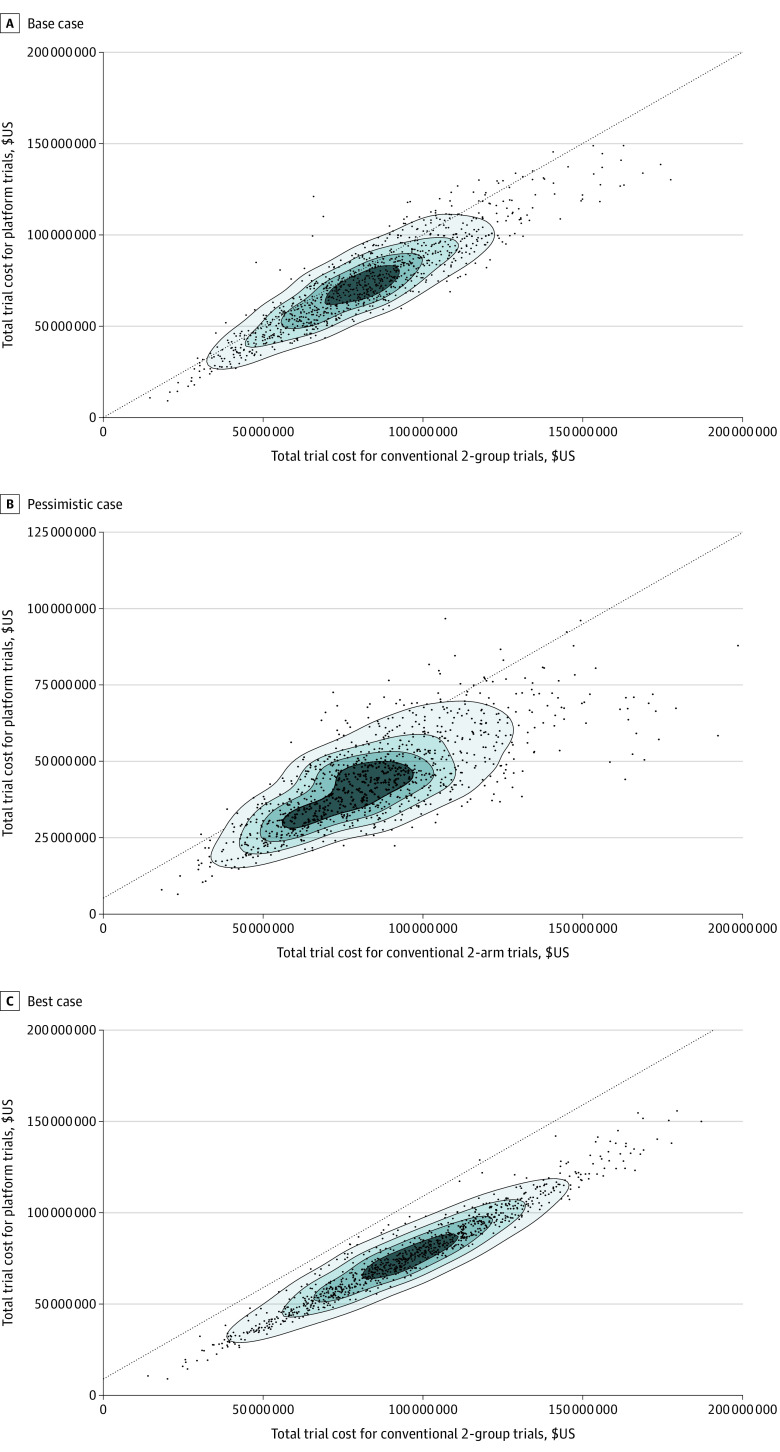
Scatterplots of the Total Trial Cost Required for Clinical Evaluation of the Subsequent 5 Interventions: A Platform Trial vs Conventional Multiple 2-Group Trials For interventions with reported treatment effects (hazard ratio [HR] and 95% CIs) on failure-free survival (FFS) and overall survival (OS), we used the point estimate as the base case, the lower CI as the best case, and the upper CI as the pessimistic case for our simulations. For the 3 intervention groups without reported results, we assumed the base case FFS and OS to have an HR of 0.75, which was the target treatment effect by the Systemic Therapy in Advancing or Metastatic Prostate Cancer: Evaluation of Drug Efficacy (STAMPEDE) investigators. The best case for these intervention groups assumed a treatment effect of 0.5625, twice the treatment effect of the target effect, for both FFS and OS, and the pessimistic case assumed that these interventions would have no treatment effects on either outcome (HR = 1.00). Dashed lines denote lines of equality.

For the subsequent interventions, we assumed that they could either be added into the platform trial or be evaluated independently using 2-group trials in either of the 2 conventional trial scenarios (Figure 4). Compared with the platform trial, the median (IQR) increase in costs for conventional trials was 12.6% (2.1%-22.6%), and 226.7% (206.8%-246.7%) for trial duration. The magnitude of the relative differences in cost and time were smaller for the subsequent interventions than the initial interventions because the platform trial had fewer active groups after starting off as a 6-group trial.^32^

As in the base case analysis, the pessimistic and best case analyses showed the platform trial having a lower total cost and cumulative trial duration than the conventional trial scenarios. In the pessimistic case that used the reported upper CIs or HR of 1.00, there were generally lower magnitudes of relative differences in cost and duration compared with the base case analysis (eTable 3 and eFigure 4 in the Supplement). In the best case analysis that was based on more optimistic treatment outcomes, there were larger magnitudes of relative differences. Since STAMPEDE used futility analyses to screen out ineffective treatments early, the pessimistic case resulted in lower sample size (eTable 4 in the Supplement) and time (eTable 5 in the Supplement).

## Discussion

In this economic evaluation, we found that combining clinical evaluation of multiple interventions into a single platform trial can substantially reduce cost and efforts rather than conducting conventional trials independently. Even with larger initial setup requirements, a platform trial can lead to important long-term efficiencies over conventional trials when there are multiple interventions that end up being evaluated in the platform. Our base case analysis showed that conducting multiple 2-group trials to evaluate up to 10 interventions can be much more costly with a median percentage increase of 57.5% than the platform trial.

There are several aspects of platform trials that lead to efficiencies. Using a common control to evaluate multiple interventions can reduce the size of the control group and, thus, overall sample size.^12^ Platform trials often use interim analyses, so there can be additional statistical efficiencies over conventional fixed designs.^6,12^ There are also operational efficiencies, with centralized site selection, patient screening, data management, study monitoring, and approval processes in a platform trial.^1,7,10,15^ Adding a new intervention into an ongoing platform requires fewer financial resources and less time than starting a new trial, but trial management and other operating cost and staffing considerations of a large platform trial should be considered when planning these potentially perpetual trials.^14,15,16^

Our findings identified several future directions. The economic evaluation framework developed for this study can be used to evaluate other adaptive trial designs and platform trials in different settings. STAMPEDE was a seamless phase 2B/3 trial where the interim and final statistical analyses were limited to concurrent control.^17,33^ There have been other trials that used nonconcurrent control,^34^ so our study has limited generalizability to these platform trials. There have also been a multitude of COVID-19 related platform trials, where it takes considerably shorter time to observe outcomes than prostate cancer.^35,36,37,38^ We did not consider the benefits of statistical inferences related to the comparative effectiveness of multiple interventions that can be made from a single trial vs trials being conducted independently. We showed that platform trials result in cost savings opportunities. The comparison of precision and validity of inferring comparative effectiveness across multiple interventions using a meta-analysis or other analysis that uses aggregate data from publications vs individual patient-level data collected through the same protocol might be an important consideration in future.

### Strengths and Limitations

The main strength of our work is the use of simulations based on a real-life example. Using STAMPEDE as an example allowed for a fair comparison of platform trial vs conventional trials with respect to number of interventions and their timing of evaluation. However, this study is subject to several limitations. We assumed that there was an infinite eligible population in our simulations, when in reality, trials often compete for participants from a limited relevant pool of patients.^39^ Different platform trials have had national-level buy-in with different stakeholders encouraging recruitment into these large trials over conventional trials, but potential recruitment benefits were not evaluated.^40^ We chose to fix the total number of interventions to be 10 across all 3 scenarios. In real life, it is possible that readouts from early clinical trial investigations could affect the decision to add an intervention into an existing platform trial or start a new trial; however, we did not consider such possibility. As costs and setup time are often not reported for conventional and platform trials,^6,20^ we chose to empirically elicit expert opinions based on a systematically compiled list of experts derived from a previous landscape analysis of master protocols.^6^ However, the survey response rate was low at 11.0%, resulting in high uncertainties. It was important to have a minimal number of questions for the survey, so we did not include an exhaustive list of items that would normally be used for trial budgeting. It is possible that consideration of other elements could have affected our results.

## Conclusions

There is a strong case for making the investment in clinical research toward a platform trial where a common infrastructure can be first built and then maintained.^8,11,12^ With increasing costs of clinical research,^20^ this can be an important solution to reduce cost and time without compromising high-quality trial evidence required to determine the most effective therapy for different clinical indications. The COVID-19 pandemic has accelerated acceptance of platform trials, but this will not be enough since the current funding models act as a hindrance to platform trials being more widely conducted.^8^

The findings of this study suggest that despite having larger initial setup requirements, consolidating clinical evaluation of multiple interventions into a single platform trial can drastically reduce cost and efforts. There is a need for a shift in thinking that can result in a more collaborative evidence-generation infrastructure with dedicated source funding to carry out platform trials.

## Notes

Supplement. **eMethods 1.** Expert Survey Questionnaire **eTable 1. **Summary of Trial Design Assumptions **eFigure 1. **Schematic Diagram of the Simulation Model **eMethods 2. **Technical Details to Survey Analyses and Cost and Time Simulations **eTable 2. **Set-Up Cost and Time Requirements for a Platform Trial Versus Multi-Group and Two-Group Trials **eFigure 2. **Set-Up Cost Comparison Between Single Platform Trial, Multigroup Trial, and 2-Group Trial **eFigure 3. **Total Cumulative Set-Up Costs Comparison Between Design Scenarios: A Single Platform Trial vs a Multigroup + 2-Group Trials and 2-Group Trials **eTable 3. **Total Cumulative Trial Duration and Costs Between Different Clinical Design Scenarios **eFigure 4. **Total Cumulative Trial Duration Comparisons Between a Platform Trial Versus Conventional Trial **eTable 4. **Estimated Sample Sizes Between Different Clinical Design Scenarios **eTable 5. **Total Cumulative Patient Follow-Up Time Between Different Clinical Design Scenarios
eReferences

## References

[1] Angus DC, Alexander BM, Berry S, ; Adaptive Platform Trials Coalition. Adaptive platform trials: definition, design, conduct and reporting considerations. Nat Rev Drug Discov. 2019;18(10):797-807. doi:10.1038/s41573-019-0034-3 31462747

[2] Park JJH, Harari O, Dron L, Lester RT, Thorlund K, Mills EJ. An overview of platform trials with a checklist for clinical readers. J Clin Epidemiol. 2020;125:1-8. doi:10.1016/j.jclinepi.2020.04.025 32416336

[3] Ventz S, Alexander BM, Parmigiani G, Gelber RD, Trippa L. Designing clinical trials that accept new arms: an example in metastatic breast cancer. J Clin Oncol. 2017;35(27):3160-3168. doi:10.1200/JCO.2016.70.1169 28530853

[4] Parmar MK, Barthel FM, Sydes M, . Speeding up the evaluation of new agents in cancer. J Natl Cancer Inst. 2008;100(17):1204-1214. doi:10.1093/jnci/djn267 18728279 PMC2528020

[5] Parmar MK, Sydes MR, Cafferty FH, . Testing many treatments within a single protocol over 10 years at MRC Clinical Trials Unit at UCL: Multi-arm, multi-stage platform, umbrella and basket protocols. Clin Trials. 2017;14(5):451-461. doi:10.1177/1740774517725697 28830236 PMC5700799

[6] Park JJH, Siden E, Zoratti MJ, . Systematic review of basket trials, umbrella trials, and platform trials: a landscape analysis of master protocols. Trials. 2019;20(1):572. doi:10.1186/s13063-019-3664-1 31533793 PMC6751792

[7] Woodcock J, LaVange LM. Master protocols to study multiple therapies, multiple diseases, or both. N Engl J Med. 2017;377(1):62-70. doi:10.1056/NEJMra1510062 28679092

[8] Park JJH, Dron L, Mills EJ. Moving forward in clinical research with master protocols. Contemp Clin Trials. 2021;106:106438. doi:10.1016/j.cct.2021.106438 34000408 PMC8120789

[9] Siden EG, Park JJ, Zoratti MJ, . Reporting of master protocols towards a standardized approach: a systematic review. Contemp Clin Trials Commun. 2019;15:100406. doi:10.1016/j.conctc.2019.100406 31334382 PMC6616543

[10] Redman MW, Allegra CJ. The master protocol concept. Semin Oncol. 2015;42(5):724-730. doi:10.1053/j.seminoncol.2015.07.009 26433553 PMC4681517

[11] Berry SM, Connor JT, Lewis RJ. The platform trial: an efficient strategy for evaluating multiple treatments. JAMA. 2015;313(16):1619-1620. doi:10.1001/jama.2015.2316 25799162

[12] Saville BR, Berry SM. Efficiencies of platform clinical trials: a vision of the future. Clin Trials. 2016;13(3):358-366. doi:10.1177/1740774515626362 26908536

[13] Cecchini M, Rubin EH, Blumenthal GM, . Challenges with novel clinical trial designs: master protocols. Clin Cancer Res. 2019;25(7):2049-2057. doi:10.1158/1078-0432.CCR-18-3544 30696689

[14] Hague D, Townsend S, Masters L, ; STAMPEDE and FOCUS4 investigators. Changing platforms without stopping the train: experiences of data management and data management systems when adapting platform protocols by adding and closing comparisons. Trials. 2019;20(1):294. doi:10.1186/s13063-019-3322-7 31138292 PMC6540437

[15] Schiavone F, Bathia R, Letchemanan K, ; STAMPEDE and FOCUS4 Trial Management Group. This is a platform alteration: a trial management perspective on the operational aspects of adaptive and platform and umbrella protocols. Trials. 2019;20(1):264. doi:10.1186/s13063-019-3216-8 31138317 PMC6540525

[16] Morrell L, Hordern J, Brown L, . Mind the gap? the platform trial as a working environment. Trials. 2019;20(1):297. doi:10.1186/s13063-019-3377-5 31138284 PMC6540560

[17] James ND, Sydes MR, Clarke NW, . Systemic therapy for advancing or metastatic prostate cancer (STAMPEDE): a multi-arm, multistage randomized controlled trial. BJU Int. 2009;103(4):464-469. doi:10.1111/j.1464-410X.2008.08034.x 18990168

[18] ClinicalTrials.gov. Systemic therapy in advancing or metastatic prostate cancer: evaluation of drug efficacy (STAMPEDE). December 22, 2005. Accessed November 1, 2021. https://clinicaltrials.gov/ct2/show/NCT00268476

[19] TCPS 2 Chapter 2: scope and approach. Canada Panel on Research Ethics. 2018. Updated September 23, 2019. Accessed June 6, 2022. https://ethics.gc.ca/eng/tcps2-eptc2_2018_chapter2-chapitre2.html#a

[20] Moore TJ, Zhang H, Anderson G, Alexander GC. Estimated costs of pivotal trials for novel therapeutic agents approved by the US Food and Drug Administration, 2015-2016. JAMA Intern Med. 2018;178(11):1451-1457. doi:10.1001/jamainternmed.2018.3931 30264133 PMC6248200

[21] Sydes MR, Parmar MKB, Mason MD, . Flexible trial design in practice—stopping arms for lack-of-benefit and adding research arms mid-trial in STAMPEDE: a multi-arm multi-stage randomized controlled trial. Trials. 2012;13:168. doi:10.1186/1745-6215-13-168 22978443 PMC3466132

[22] Sydes MR, Parmar MKB, James ND, . Issues in applying multi-arm multi-stage methodology to a clinical trial in prostate cancer: the MRC STAMPEDE trial. Trials. 2009;10:39. doi:10.1186/1745-6215-10-39 19519885 PMC2704188

[23] James ND, Spears MR, Clarke NW, ; STAMPEDE Investigators. Failure-free survival and radiotherapy in patients with newly diagnosed nonmetastatic prostate cancer: data from patients in the control arm of the STAMPEDE trial. JAMA Oncol. 2016;2(3):348-357. doi:10.1001/jamaoncol.2015.4350 26606329 PMC4789485

[24] Mason MD, Clarke NW, James ND, ; STAMPEDE Investigators. Adding celecoxib with or without zoledronic acid for hormone-naïve prostate cancer: long-term survival results from an adaptive, multiarm, multistage, platform, randomized controlled trial. J Clin Oncol. 2017;35(14):1530-1541. doi:10.1200/JCO.2016.69.0677 28300506 PMC5455701

[25] James ND, de Bono JS, Spears MR, ; STAMPEDE Investigators. Abiraterone for prostate cancer not previously treated with hormone therapy. N Engl J Med. 2017;377(4):338-351. doi:10.1056/NEJMoa1702900 28578639 PMC5533216

[26] Parker CC, James ND, Brawley CD, ; Systemic Therapy for Advanced or Metastatic Prostate cancer: Evaluation of Drug Efficacy (STAMPEDE) Investigators. Radiotherapy to the primary tumour for newly diagnosed, metastatic prostate cancer (STAMPEDE): a randomised controlled phase 3 trial. Lancet. 2018;392(10162):2353-2366. doi:10.1016/S0140-6736(18)32486-3 30355464 PMC6269599

[27] Tarricone R. Cost-of-illness analysis: what room in health economics? Health Policy. 2006;77(1):51-63. doi:10.1016/j.healthpol.2005.07.016 16139925

[28] Polsky D, Glick H. Costing and cost analysis in randomized controlled trials: caveat emptor. Pharmacoeconomics. 2009;27(3):179-188. doi:10.2165/00019053-200927030-00001 19354338 PMC2971527

[29] Hozo SP, Djulbegovic B, Hozo I. Estimating the mean and variance from the median, range, and the size of a sample. BMC Med Res Methodol. 2005;5:13. doi:10.1186/1471-2288-5-13 15840177 PMC1097734

[30] Meyer EL, Mesenbrink P, Mielke T, Parke T, Evans D, König F; EU-PEARL (EU Patient-cEntric clinicAl tRial pLatforms) Consortium. Systematic review of available software for multi-arm multi-stage and platform clinical trial design. Trials. 2021;22(1):183. doi:10.1186/s13063-021-05130-x 33663579 PMC7931508

[31] Wathen JK. OCTOPUS: optimize clinical trials on platforms using simulation update. 2021. Accessed November 2, 2021. https://kwathen.github.io/OCTOPUS/

[32] Sydes MR, Spears MR, Mason MD, ; STAMPEDE Investigators. Adding abiraterone or docetaxel to long-term hormone therapy for prostate cancer: directly randomised data from the STAMPEDE multi-arm, multi-stage platform protocol. Ann Oncol. 2018;29(5):1235-1248. doi:10.1093/annonc/mdy072 29529169 PMC5961425

[33] Sydes MR, James ND, Mason MD, . Flexible trial design in practice—dropping and adding arms in STAMPEDE: a multi-arm multi-stage randomised controlled trial. Trials. 2011;12(suppl 1). doi:10.1186/1745-6215-12-S1-A3 PMC3466132 22978443

[34] Barker AD, Sigman CC, Kelloff GJ, Hylton NM, Berry DA, Esserman LJ. I-SPY 2: an adaptive breast cancer trial design in the setting of neoadjuvant chemotherapy. Clin Pharmacol Ther. 2009;86(1):97-100. doi:10.1038/clpt.2009.68 19440188

[35] Angus DC, Derde L, Al-Beidh F, ; Writing Committee for the REMAP-CAP Investigators. Effect of hydrocortisone on mortality and organ support in patients with severe COVID-19: the REMAP-CAP COVID-19 corticosteroid domain randomized clinical trial. JAMA. 2020;324(13):1317-1329. doi:10.1001/jama.2020.17022 32876697 PMC7489418

[36] Horby P, Lim WS, Emberson JR, ; RECOVERY Collaborative Group. Dexamethasone in hospitalized patients with COVID-19. N Engl J Med. 2021;384(8):693-704. doi:10.1056/NEJMoa2021436 32678530 PMC7383595

[37] Yu LM, Bafadhel M, Dorward J, ; PRINCIPLE Trial Collaborative Group. Inhaled budesonide for COVID-19 in people at high risk of complications in the community in the UK (PRINCIPLE): a randomised, controlled, open-label, adaptive platform trial. Lancet. 2021;398(10303):843-855. doi:10.1016/S0140-6736(21)01744-X 34388395 PMC8354567

[38] Reis G, Moreira Silva EADS, Medeiros Silva DC, ; TOGETHER Investigators. Effect of early treatment with hydroxychloroquine or lopinavir and ritonavir on risk of hospitalization among patients with COVID-19: the TOGETHER randomized clinical trial. JAMA Netw Open. 2021;4(4):e216468. doi:10.1001/jamanetworkopen.2021.6468 33885775 PMC8063069

[39] Gelinas L, Lynch HF, Bierer BE, Cohen IG. When clinical trials compete: prioritising study recruitment. J Med Ethics. 2017;43(12):803-809. doi:10.1136/medethics-2016-103680 28108613 PMC5519451

[40] National Institute for Health and Care Research. Recruiting patients for clinical trials for COVID-19 therapeutics. 2020. Accessed August 25, 2021. https://www.nihr.ac.uk/news/uks-chief-medical-officers-urge-hospitals-to-recruit-60-of-eligible-covid-19-patients-into-recovery-trial/25515

