# Supplementary material for: Economic Evaluation of Cost and Time Required for a Platform Trial vs Conventional Trials
Source: JAMA Netw Open. 2022 Jul 12;5(7):e2221140. doi: 10.1001/jamanetworkopen.2022.21140 (PMC9277502; doi:10.1001/jamanetworkopen.2022.21140)
Supplement: Supplement. — eMethods 1. Expert Survey Questionnaire eTable 1. Summary of Trial Design Assumptions eFigure 1. Schematic Diagram of the Simulation Model eMethods 2. Technical Details to Survey Analyses and Cost and Time Simulations eTable 2. Set-Up Cost and Time Requirements for a Platform Trial Versus Multi-Group and Two-Group Trials eFigure 2. Set-Up Cost Comparison Between Single Platform Trial, Multigroup Trial, and 2-Group Trial eFigure 3. Total Cumulative Set-Up Costs Comparison Between Design Scenarios: A Single Platform Trial vs a Multigroup + 2-Group Trials and 2-Group Trials eTable 3. Total Cumulative Trial Duration and Costs Between Different Clinical Design Scenarios eFigure 4. Total Cumulative Trial Duration Comparisons Between a Platform Trial Versus Conventional Trial eTable 4. Estimated Sample Sizes Between Different Clinical Design Scenarios eTable 5. Total Cumulative Patient Follow-Up Time Between Different Clinical Design Scenarios eReferences [file jamanetwopen-e2221140-s001.pdf]

Supplemental Online Content

Park JJH, Sharif B, Harari O, et al. Economic evaluation of cost and time required for a platform trial vs conventional trials. *JAMA Netw Open*. 2022;5(7):e2221140. doi:10.1001/jamanetworkopen.2022.21140

eMethods 1. Expert Survey Questionnaire
eTable 1. Summary of Trial Design Assumptions
eFigure 1. Schematic Diagram of the Simulation Model
eMethods 2. Technical Details to Survey Analyses and Cost and Time Simulations
eTable 2. Set-Up Cost and Time Requirements for a Platform Trial Versus Multi-Group and Two-Group Trials
eFigure 2. Set-Up Cost Comparison Between Single Platform Trial, Multigroup Trial, and 2-Group Trial
eFigure 3. Total Cumulative Set-Up Costs Comparison Between Design Scenarios: A Single Platform Trial vs a Multigroup + 2-Group Trials and 2-Group Trials
eFigure 4. Total Cumulative Trial Duration Comparisons Between a Platform Trial Versus Conventional Trial
eTable 3. Total Cumulative Trial Duration and Costs Between Different Clinical Design Scenarios
eTable 4. Estimated Sample Sizes Between Different Clinical Design Scenarios
eTable 5. Total Cumulative Patient Follow-Up Time Between Different Clinical Design Scenarios
eReferences

This supplemental material has been provided by the authors to give readers additional information about their work.

© 2022 Park JJH et al. *JAMA Network Open.*

eMethods 1: Expert Survey Questionnaire

Instructions:

The questions in this survey involve the costs and time required to conduct randomized clinical trials (RCTs). The responses will be used to compare the efficiency of different approaches in conducting clinical trials: 1) 2-arm trials; 2) multi-arm trials; and 3) platform trials.

First, we are considering traditional 2-arm trials with one experimental intervention arm being compared to the control arm. Second, we consider multi-arm trials, where there are two or more experimental intervention arms being compared against the control arm. Lastly, we are considering platform trials, a relatively newer type of randomized clinical trial designs that allow simultaneous comparison of multiple interventions against a common control using a pre-specified interim analyses plan. This single overarching protocol called a master (or core) protocol dictates how new interventions are introduced after the trial is initiated, thus allowing for multiple interventions to be evaluated in a perpetual manner.

This survey will begin by asking general questions about your area of expertise, current employment sector, and role in clinical trial related research. The part of the questionnaire regarding costs is broken into three parts of 1) Trial setup; 2) Trial conduct; and 3) Trial analyses.

This survey is not intended to be exhaustive. It is intended to identify key costing items that will allow us to compare different clinical trial evaluation approaches to each other.

Please try to answer all questions so that we can compare the costs of different clinical trials fairly.

General information:

1) Please indicate the country of your residence (e.g. US, UK)?

2) Please indicate your PRIMARY role in clinical trials related research
   a) Clinical investigator
   b) Health economist
   c) Journal editor
   d) Regulatory assessor
   e) Research ethicist
   f) Research funding board/panel member
   g) Trial manager
   h) Trial methodologist
   i) Other: Please specify _____

3) Please indicate your CURRENT employment sector. Select all that apply.
   a) Public sector
   b) Industry
   c) Philanthropy
   d) Regulatory agency
   e) Other: Please specify_____

4) Please indicate therapeutic area(s) that you have experience in. Select ALL that apply
   - Cardiology
   - Dermatology
   - Gastroenterology
   - Hematology

© 2022 Park JJH et al. *JAMA Network Open.*

- Immunology
- Infectious diseases
- Metabolism and endocrinology
- Neurology
- Oncology
- Psychiatry
- Rheumatology
- Urology
- Other: Please specify ______

5) Please indicate your experience in clinical trial research. Select ALL that apply
   a) Trial protocol development
   b) Trial approval submission (e.g. ethics and regulatory approval submission)
   c) Trial database development and/or management
   d) Trial operation planning
   e) Trial conduct and reporting
   f) Trial budgeting
   g) Ethics and/or regulatory assessment
   h) Statistical analyses and/or data safety monitoring board (DSMB)

Trial set-up:

The following questions are aimed to gather broad estimates across all therapeutic areas and for most situations.

Time required to develop a trial protocol (in months)

1) In your opinion, what is the time required to develop a study trial protocol for the following clinical trials?

	2-arm trial	Multi-arm trial	Platform trial
Average (months)			
Minimum (months)			
Maximum (months)			

Cost required to develop a trial protocol (in 2021 USD)

2) In your opinion, what is the cost required to develop a study trial protocol for the following clinical trials?

	2-arm trial	Multi-arm trial	Platform trial
Average ($)			
Minimum ($)			
Maximum ($)			

Time required to obtain study approval (in months)

3) In your opinion, what is the time required to obtain study approvals for the following clinical trials?

	2-arm trial	Multi-arm trial	Platform trial
Average (months)			
Minimum (months)			
Maximum (months)			

Cost required to obtain study approval (in 2021 USD)

© 2022 Park JJH et al. *JAMA Network Open.*

4) In your opinion, what is the cost required to obtain study approvals for the following clinical trials?

	2-arm trial	Multi-arm trial	Platform trial
Average ($)			
Minimum ($)			
Maximum ($)			

Time required for database development (in months)

5) In your opinion, what is the time required to set up a database for the following clinical trials?

	2-arm trial	Multi-arm trial	Platform trial
Average (months)			
Minimum (months)			
Maximum (months)			

Cost required for database development (in 2021 USD)

6) In your opinion, what is the cost required to set up a database for the following clinical trials?

	2-arm trial	Multi-arm trial	Platform trial
Average ($)			
Minimum ($)			
Maximum ($)			

Cost required to set up clinical trial sites (in 2021 USD)

7) In your opinion, what is the cost required to set up a single clinical trial site for a clinical trial?

	Clinical trial
Average ($/site)	
Minimum ($/site)	
Maximum ($/site)	

© 2022 Park JJH et al. *JAMA Network Open.*

Trial conduct:

Please estimate the cost required for conducting the trial. These survey questions are aimed to gather broad estimates across all therapeutic areas.

Cost for patient recruitment (monthly cost per patient in 2021 USD)

8) What is the **monthly cost required to recruit a single patient** for a clinical trial?

	Clinical trial
Average (monthly cost for per patient)	
Minimum (monthly cost for per patient)	
Maximum (monthly cost for per patient)	

Cost for patient follow-up (monthly cost per patient in 2021 USD)

9) What is the **monthly cost required to follow-up on a single patient** for the following scenarios?

	Clinical trial
Average (monthly cost per patient)	
Minimum (monthly cost per patient)	
Maximum (monthly cost per patient)	

Cost for clinical trial site management (monthly cost per site in 2021 USD)

10) In your opinion, what is the **monthly cost required to manage a single clinical trial** for the following scenarios?

	Clinical Trial
Average (monthly cost for per site)	
Minimum (monthly cost for per site)	
Maximum (monthly cost for per site)	

Cost required for clinical trial database management (monthly cost in 2021 USD)

11) In your opinion, what is the **monthly cost required to manage a database for the following scenarios**?

	2-arm trial	Multi-arm trial	Platform trial
Average (monthly cost)			
Minimum (monthly cost)			
Maximum (monthly cost)			

Time required for adding new arms (in months)

12) In your opinion, how much **time is required to add a new intervention arm during a platform trial**?

	Platform Trial
Average (months)	
Minimum (months)	
Maximum (months)	

Cost required for adding new arms (in 2021 USD)

13) In your opinion, what is the **cost required to add a new intervention arm during a platform trial**?

	Platform Trial
Average ($)	
Minimum ($)	
Maximum ($)	

© 2022 Park JJH et al. *JAMA Network Open.*

Trial analyses:

Cost required for interim analyses (in 2021 USD)

14) In your opinion, what is the **cost required to conduct a single INTERIM analysis for a single intervention in the following clinical trials** (2021 USD)?

	2-arm trial	Multi-arm trial	Platform trial
Average ($)			
Minimum ($)			
Maximum ($)			

Cost required for final analysis (in 2021 USD)

15) In your opinion, what is the **cost required to conduct the FINAL analysis for a single intervention in the following clinical trials** (2021 USD)?

	2-arm trial	Multi-arm trial	Platform trial
Average ($)			
Minimum ($)			
Maximum ($)			

© 2022 Park JJH et al. *JAMA Network Open.*

eTable 1. Summary of Trial Design Assumptions

Parameter	Assumption	Source
Number of sites in a platform trial	120	Sydes et al. Trials 2012, 13:168[1]
Number of sites in a multi-arm trial	80	Assumed
Number of sites in a two-arm trial	50	Assumed
Annual recruitment rate per site	4.167	Annual recruitment rate calculated based on published protocol of STAMPEDE (500 patients per year / 120 = 4.167 per site)
Randomization ratio in a platform trial for intervention 1-5	Unequal allocation in favor of the control (2:1)	Sydes et al. Trials 2012, 13:168[1]
Randomization ratio in a platform trial for intervention 6-10	Equal allocation (1:1)	
Randomization ratio in a multi-arm trial for intervention 1-5	Unequal allocation in favor of the control (2:1)	Assumed to be the same as STAMPEDE
Randomization ratio in a two-arm trial for intervention 1-10	Equal allocation (1:1)	Assumed
Maximum sample size for an intervention	443	Assumed for intervention 1-10
Maximum sample size for the control in platform or multi-arm trial for evaluation of intervention 1-5	886	Assumed for concurrent controls for intervention 1-5
Maximum sample size for the control in platform trial for evaluation of intervention 6-10	443	Assumed for concurrent control for intervention 1-10
Maximum sample size for the control in two-arm trial	443	Assumed
Start time of for the first five interventions (intervention 1-5)	0	Relative time calculated from the start date of STAMPEDE[1] (2005-10-05); Assumed to be equal for all scenarios
Start time (months) for intervention 6	72.2	
Start time (months) for intervention 7	87.4	
Start time (months) for intervention 8	104.6	
Start time (months) for intervention 9	127.6	
Start time (months) for intervention 10	140.0	
Statistical analysis	Log rank test based on pairwise analysis versus concurrent control	Sydes et al. Trials 2012, 13:168;[1] Assumed to be equal for all scenarios
Event rates assumptions	Median failure free survival (FFS) of 24 months and overall survival (OS) of 48 months	Sydes et al. Trials 2012, 13:168;[1] Assumed to be equal for all scenarios
Interim analysis 1 time	114 FFS events observed in concurrent control	Sydes et al. Trials 2012, 13:168[1]
Interim analysis 2 time	215 FFS events observed in concurrent control	Sydes et al. Trials 2012, 13:168[1]
Interim analysis 3 time	334 FFS events observed in concurrent control	Sydes et al. Trials 2012, 13:168[1]
Final analysis time	400 deaths observed in concurrent control	Sydes et al. Trials 2012, 13:168[1]
Decision rule for futility at interim analysis 1	FFS HR of 1.0 or greater	Sydes et al. Trials 2012, 13:168[1]
Decision rule for futility at interim analysis 2	FFS HR of 0.92 or greater	Sydes et al. Trials 2012, 13:168[1]
Decision rule for futility at interim analysis 2	FFS HR of 0.89 or greater	Sydes et al. Trials 2012, 13:168[1]
Effect sizes for intervention 1	Reported FFS HR (95% CI): 0.92 (0.81, 1.04)<br>Reported OS HR (95% CI): 0.94 (0.79, 1.11)	James et al 2016 Lancet 2016; 387: 1163–77[2]
Effect sizes for intervention 2	Reported FFS HR (95% CI): 0.61 (0.53, 0.70)<br>Reported OS HR (95% CI): 0.78 (0.66, 0.93)	James et al 2016 Lancet 2016; 387: 1163–77[2]
Effect sizes for intervention 3	Reported FFS HR (95% CI): 0.87 (0.74, 1.03)<br>Reported OS HR (95% CI): 0.98 (0.80, 1.20)	Mason 2017 J Clin Oncol 35:1530-1541. [3]
Effect sizes for intervention 4	Reported FFS HR (95% CI): 0.62 (0.54, 0.70)<br>Reported OS HR (95% CI): 0.82 (0.69, 0.97)	James et al 2016 Lancet 2016; 387: 1163–77[2]
Effect sizes for intervention 5	Reported FFS HR (95% CI): 0.84 (0.72, 0.99)<br>Reported OS HR (95% CI): 0.86 (0.70, 1.05)	Mason 2017 J Clin Oncol 35:1530-1541. [3]
Effect sizes for intervention 6	Reported FFS HR (95% CI): 0.29 (0.25, 0.34)<br>Reported OS HR (95% CI): 0.63 (0.52, 0.76)	James 2017 N Engl J Med 2017;377:338-51.[4]
Effect sizes for intervention 7	Reported FFS HR (95% CI): 0.76 (0.68, 0.84)<br>Reported OS HR (95% CI): 0.92 (0.80, 1.06)	Parker 2018 The Lancet. 2018 1;392(10162):2353-66.[5]
Effect sizes for intervention 8-10	FFS and OS HRs of 0.75 (base case)<br>FFS and OS HR of 1.00 (worst case)<br>FFS and OS HR of 0.5625 (best case)	• Assumed as effect sizes not reported at the time of this study<br>• Target effect size used as base case from James et al. Lancet Oncol. 13: 549-58<br>• Best case assumes twice as large effect size as the base case on the log scale

© 2022 Park JJH et al. *JAMA Network Open.*

eFigure 1. Schematic Diagram of the Simulation Model

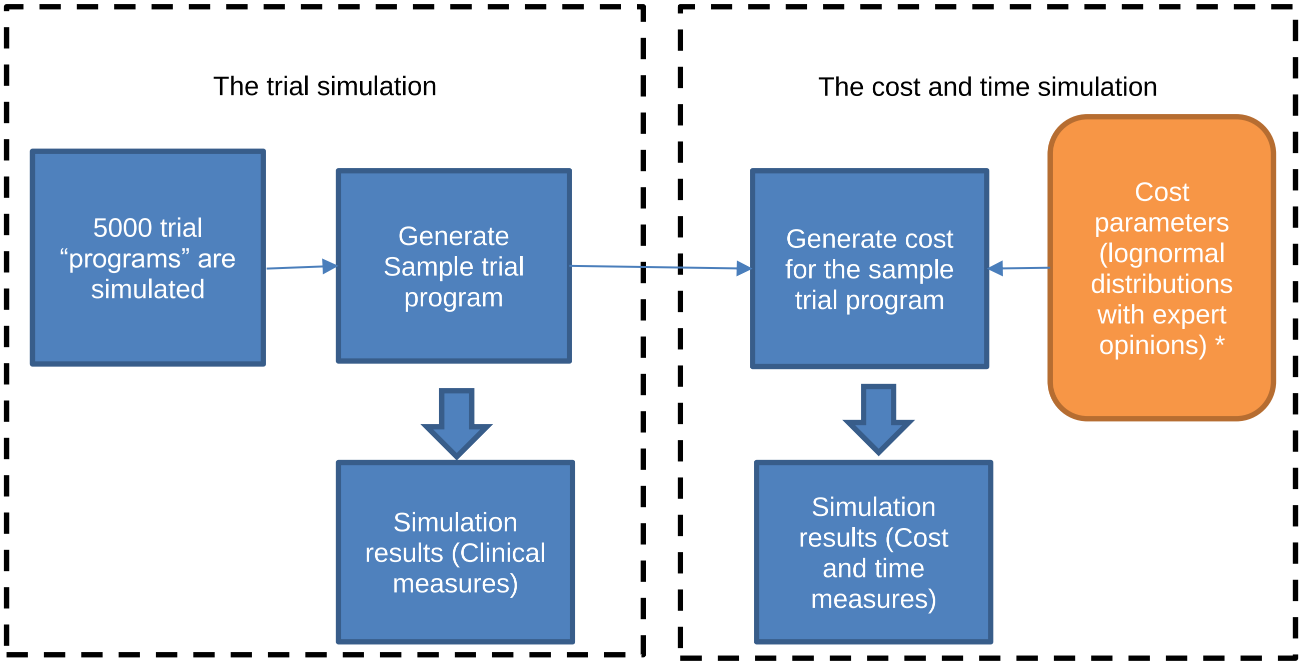

© 2022 Park JJH et al. *JAMA Network Open.*

eMethods 2: Technical Details to Survey Analyses and Cost and Time Simulations

Survey analyses

Given the mean estimate and range (R) from the expert opinion: if data not normally distributed (cost parameters) then S=R/6 (S is the standard deviation of the distribution that we need in Step 2) , according to the Chebyshev's inequality: $(P(|X-\mu| < k\sigma) \geq 1 - \frac{1}{k^2})$, which results in the range covers approximately 6σ, i.e., $\sigma \approx \frac{R}{6}$. If our data are normally distributed, then P[-2σ <X - μ < 2σ] = 0.95, and therefore, the range covers approximately 4σ, i.e., $\sigma \approx \frac{R}{4}$. Using Sigma (S) from above, R as the range from experts and (x-hat) as the mean from expert opinion, below is the equations form method of moments to use for calculating mean and standard deviation of lognormal (Cost parameters) and normal (time parameters):[6]

- Cost parameters: Lognormal distribution:

$$\mu = \ln \bar{x} - \frac{1}{2}\ln\left(\frac{s^2}{\bar{x}^2} + 1\right) = \ln\left(\bar{x} \Big/ \sqrt{\frac{s^2}{\bar{x}^2} + 1}\right) \qquad \sigma^2 = \ln\left(\frac{s^2}{\bar{x}^2} + 1\right)$$

- Time parameters: Normal distribution- since P[-2σ <X - μ < 2σ] = 0.95, and therefore, the range covers approximately 4σ, i.e., $\sigma \approx \frac{R}{4}$.

Costing approach

We used a mixed costing approach in estimating cost of a trial. As shown, we calculated the setup cost including protocol, IRB approval, database-management, and sites in addition to recruitment cost. We next calculated the conduct cost including cost per patient follow-up for both treatment and control arms. Finally, the analysis cost for final and interim analysis were assigned.

Each sample trial simulation from OCTOPUS is used as in input to the trial cost simulator in which cost parameters associated with various resource use are utilized together with resulted sample size, number of sites and number and duration of treatments in a trial to calculate total cost of the trial. The results were then recorded for 1,000 simulations iterations (for 5 scenarios for the total of 5,000) which result in a distribution of total cost of each trial design.

The model calculates cost of each trial program by generating a cost parameter sample from the distributions of cost parameters (all lognormal distributed) and multiplying by number of patient visits (for sample-size based parameters from OCTOPUS) and number of sites/region (for fixed cost parameters). The total cost for each trial program is the summation of resource use cost for the number of patients screened, treated, and monitored, in addition to treatment costs and fixed costs for the total duration of the simulated trial program. Finally, the distribution of cost for a total of 5,000 trials programs will be constructed and reported for each trial design.

© 2022 Park JJH et al. *JAMA Network Open.*

Trial Cost Function Equation

Setup costs	$Total\ cost\ per\ trial =$ $+C_{protocol} + C_{irb} + C_{database\_management} + n_{sites} * C_{site}$ $+ n_{controls} * c_{per\ patient/recruitement}$
Trial conduct costs	$+ n_{controls} * c_{per\ patient/followup} * Total_{duration}$ $+ \sum_{ISA} (n_{ISA}\ *\ c_{recruitement\ per\ patient}) +$ $(+ n_{controls}\ *\ c_{per\frac{patient}{followup}}\ *\ follow\ up\_time)$ $(+ n_{controls}\ * c_{per\frac{patient}{followup}} * follow\ up\_time)$
Analysis costs	$+ C_{final\ analysis} + n_{interim\ analysis} * C_{interim\ analysis}$

© 2022 Park JJH et al. *JAMA Network Open.*

eTable 2. Set-Up Cost and Time Requirements for a Platform Trial Versus Multi-Group and Two-Group Trials

Cost (million USD) and time (years) requirements	Scenario 1: A platform trial Mean (SD)	Scenario 2: A multi-arm + two-arm trials Mean (SD)	Scenario 3: Two-arm trials Mean (SD)	Scenario 2 vs 1		Scenario 3 vs 1	
				Mean difference (SD)	% difference Median (IQR)	Mean difference (SD)	% difference Median (IQR)
**Set-up requirement for a single trial**							
Set-up cost of a single trial	2.24 (1.222)	1.59 (0.82)	1.09 (0.51)	-0.65(0.41)	-26.73% (-30.80%; -25.41%)	-1.15 (0.71)	-48.10% (-53.47%; -46.20%)
Set-up time of a single trial	1.305 (0.453)	0.78 (0.25)	0.65 (0.22)	-0.52(0.52)	-28.18% (-55.47%; -15.59%)	-0.65(0.5)	-40.80% (-64.09%; -29.78%)
**Cumulative set-up requirements for clinical evaluation of 10 interventions**							
Cumulative set-up cost	2.24 (1.222)	7.044 (3.383)	10.902 (5.138)	4.803 (2.167)	216.7% (202.2%; 242.4%)	8.662 (3.923)	391.1% (365.3%; 437.9%)
Cumulative trial set-up time	1.31 (0.45)	4.05 (1.12)	6.52 (2.2)	2.74 (1.21)	208.5% (133.1%; 330.7%)	5.22 (2.25)	398.6% (259.3%; 601.5%)

© 2022 Park JJH et al. *JAMA Network Open.*

eFigure 2. Set-Up Cost Comparison Between Single Platform Trial, Multigroup Trial, and 2-Group Trial

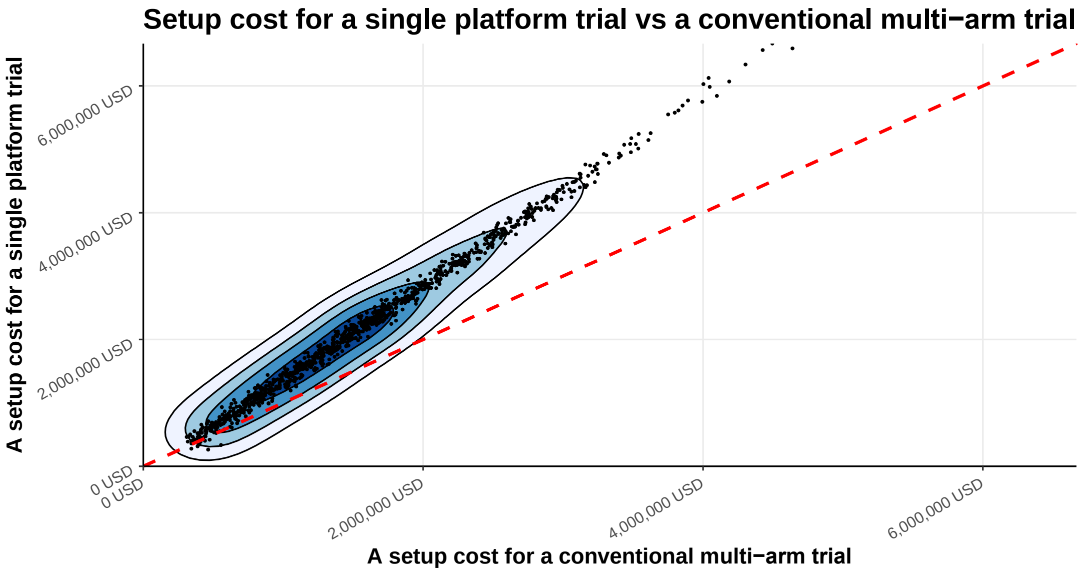

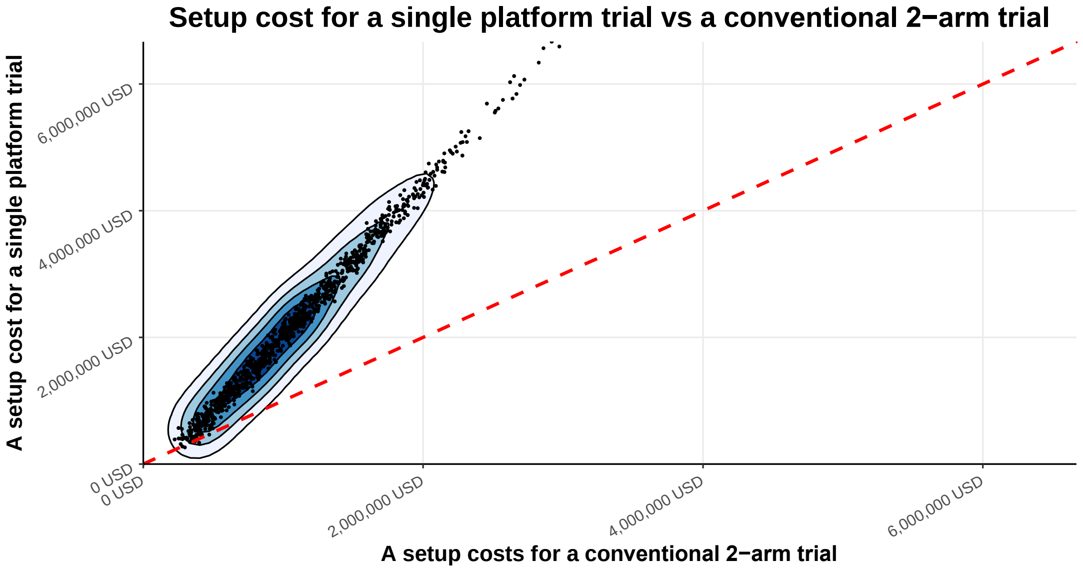

© 2022 Park JJH et al. *JAMA Network Open.*

eFigure 3. Total Cumulative Set-Up Costs Comparison Between Design Scenarios: A Single Platform Trial vs a Multigroup Plus 2-Group Trials and 2-Group Trials

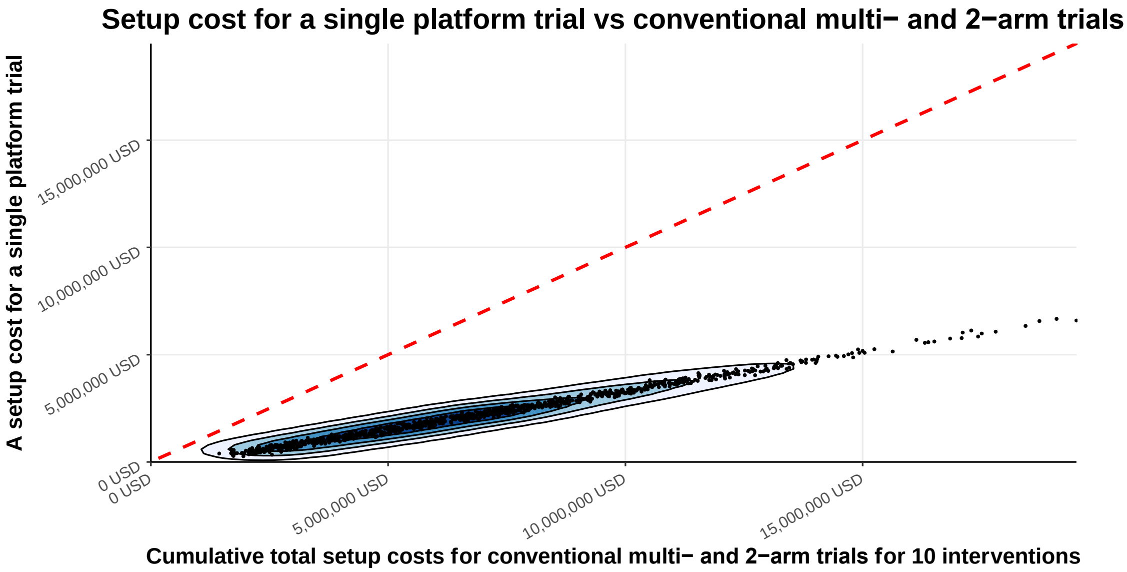

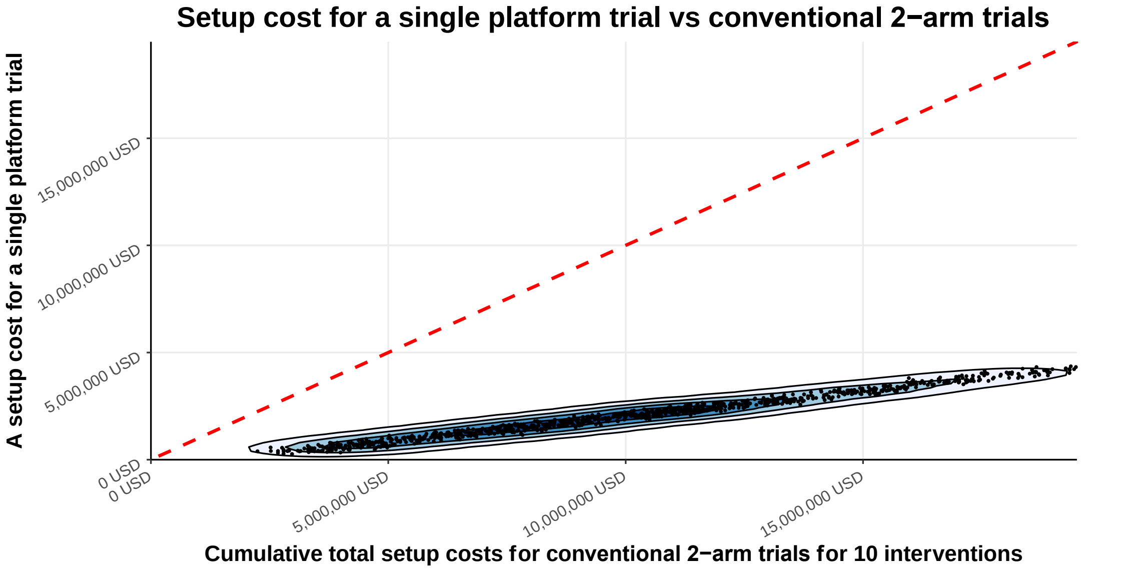

© 2022 Park JJH et al. *JAMA Network Open.*

eTable 3. Total Cumulative Trial Duration and Costs Between Different Clinical Design Scenarios

Cost (million USD) and time (years) requirements	Scenario 1: A platform trial Mean (SD)	Scenario 2: A multi-arm + two-arm trials Mean (SD)	Scenario 3: Two-arm trials Mean (SD)	Scenario 2 vs 1		Scenario 3 vs 1	
				Mean difference (SD)	% difference Median (IQR)	Mean difference (SD)	% difference Median (IQR)
**Base case**							
Total cumulative trial duration for all 10 interventions (1-10)	20.75 (1.16)	55.96 (3.38)	85.85 (8.56)	35.21 (3.52)	171.1% (158.3%; 184.3%)	65.09 (8.58)	311.9% (282%; 349.1%)
Total cumulative trial duration for first five interventions (1-5)	5.96 (0.19)	8.1 (0.58)	37.99 (7.48)	2.14 (0.61)	36.1% (30.8%; 41.5%)	32.03 (7.48)	526.3% (429.4%; 657.9%)
Total cumulative trial duration for last five interventions (6-10)	14.74 (1.16)	47.86 (3.35)	47.86 (3.35)	33.12 (3.49)	226.7% (206.8%; 246.7%)	33.12 (3.49)	226.7% (206.8%; 246.7%)
Total trial cost for all interventions (1-10)	104.951 (32.512)	122.105 (36.744)	163.403 (51.042)	17.154 (10.569)	17.4% (12.1%; 22.5%)	58.452 (24.942)	57.7% (43.1%; 69.9%)
Total trial cost for first five treatments (1-5)	31.356 (9.022)	40.403 (15.277)	81.701 (25.521)	9.047 (10.159)	28% (5.5%; 50.1%)	50.345 (17.957)	158.4% (136.9%; 184.1%)
Total trial cost for last five treatments (6-10)	73.594 (23.893)	81.701 (25.521)	81.701 (25.521)	8.107 (10.6)	12.6% (2.1%; 22.6%)	8.107 (10.6)	12.6% (2.1%; 22.6%)
Trial management cost for evaluation of first five interventions (1-5)	3.876 (2.035)	3.155 (1.67)	7.756 (5.324)	-0.722 (0.431)	-18.3% (-21.1%; -15.9%)	3.88 (4.059)	92.7% (11.7%; 198.2%)
Trial management cost for evaluation of last five interventions (6-10)	8.638 (4.613)	7.756 (5.324)	7.756 (5.324)	-0.882 (3.696)	-14.1% (-45.8%; 30%)	-0.882 (3.696)	-14.1% (-45.8%; 30%)
**Pessimistic case**							
Total cumulative trial time for all 10 interventions (1-10)	15.95 (1.53)	39.17 (5.96)	58.47 (7.38)	23.21 (6.17)	144.2% (118.3%; 173.4%)	42.52 (7.45)	266% (232.6%; 303.8%)
Total cumulative trial time for first five interventions (1-5)	5.9 (0.53)	7.29 (1.43)	26.6 (4.18)	1.4 (1.51)	26.9% (21.1%; 32.6%)	20.7 (4.21)	334.5% (298.9%; 389.9%)
Total cumulative trial time for first five interventions (6-10)	9.94 (1.53)	31.87 (5.78)	31.87 (5.78)	21.93 (5.99)	220.4% (176.5%; 271.3%)	21.93 (5.99)	220.4% (176.5%; 271.3%)
Total trial cost for all interventions (1-10)	70.945 (21.549)	80.254 (26.483)	101.967 (32.599)	9.309 (16.519)	13% (-1.8%; 27.9%)	31.022 (20.27)	43.8% (25.7%; 63.9%)
Total trial cost for first five treatments + concurrent control	27.123 (7.977)	29.27 (12.571)	50.984 (16.299)	2.147 (9.989)	5.9% (-14.9%; 27.8%)	23.861 (11.333)	82.8% (62.4%; 107.3%)
Total trial cost for last five treatments + concurrent control	43.822 (14.605)	50.984 (16.299)	50.984 (16.299)	7.162 (11.035)	17.1% (1.1%; 36%)	7.162 (11.035)	17.1% (1.1%; 36%)
Trial management cost for evaluation of first five interventions (1-5)	3.876 (2.035)	2.811 (1.643)	4.704 (2.945)	-1.065 (0.824)	-24.3% (-27.3%; -21.5%)	0.827 (1.723)	2% (-2.8%; 40.8%)
Trial management cost for evaluation of last five interventions (6-10)	5.54 (3.037)	4.704 (2.945)	4.704 (2.945)	-0.836 (1.91)	-21% (-33.5%; -2.5%)	-0.836 (1.91)	-21% (-33.5%; -2.5%)

© 2022 Park JJH et al. *JAMA Network Open.*

Cost (million USD) and time (years) requirements	Scenario 1: A platform trial Mean (SD)	Scenario 2: A multi-arm + two-arm trials Mean (SD)	Scenario 3: Two-arm trials Mean (SD)	Scenario 2 vs 1		Scenario 3 vs 1	
				Mean difference (SD)	% difference Median (IQR)	Mean difference (SD)	% difference Median (IQR)
**Best case**							
Total cumulative trial time for all 10 interventions (1-10)	20.83 (1.03)	57.17 (1.95)	96.25 (5.42)	36.33 (2.18)	175.4% (164.4%; 186%)	75.42 (5.48)	365.6% (345.4%; 386.8%)
Total cumulative trial time for first five interventions (1-5)	6.03 (0.21)	8.37 (0.37)	47.46 (4.74)	2.34 (0.42)	38.7% (33.9%; 44%)	41.42 (4.74)	703.3% (673%; 733.8%)
Total cumulative trial time for first five interventions (6-10)	14.82 (1.03)	48.79 (1.9)	48.79 (1.9)	33.98 (2.13)	230.8% (213.8%; 248.7%)	33.98 (2.13)	230.8% (213.8%; 248.7%)
Total trial cost for all interventions (1-10)	111.02 (34.322)	131.328 (39.654)	190.476 (58.02)	20.308 (7.654)	18.6% (15.2%; 22.2%)	79.456 (25.584)	73.5% (67.8%; 78.7%)
Total trial cost for first five treatments + concurrent control	34.526 (9.938)	36.09 (12.426)	95.238 (29.01)	1.564 (6.476)	0.7% (-6.4%; 9.8%)	60.712 (19.73)	175.3% (160.8%; 189.8%)
Total trial cost for last five treatments + concurrent control	76.494 (24.69)	95.238 (29.01)	95.238 (29.01)	18.744 (7.574)	25.9% (21%; 31.3%)	18.744 (7.574)	25.9% (21%; 31.3%)
Trial management cost for evaluation of first five interventions (1-5)	3.876 (2.035)	3.265 (1.717)	11.67 (6.492)	-0.612 (0.35)	-15.7% (-17.7%; -13.6%)	7.794 (4.652)	215.8% (203%; 226.7%)
Trial management cost for evaluation of last five interventions (6-10)	8.71 (4.612)	11.67 (6.492)	11.67 (6.492)	2.96 (2.858)	40.2% (30.9%; 48.7%)	2.96 (2.858)	40.2% (30.9%; 48.7%)

The estimated total duration included time required to set-up and conduct times. The total trial cost included all costs required to set up and conduct the trial.

For interventions with reported treatment effects (hazard ratio [HR] and 95% confidence intervals) on FFS and OS, we used the point estimate as the ‘base case’, the lower confidence interval as the ‘best case’, and the upper confidence interval as the ‘pessimistic case’ for our simulations. For the last three intervention arms without reported results, we assumed the base case FFS and OS to have HR of 0.75, which was the target treatment effects by the STAMPEDE investigators. The best case scenario for these intervention arms assumed a treatment effect of 0.5625, twice as large treatment effects as the target effects, for both FFS and OS, and the pessimistic case scenario assumed that these interventions would have no treatment effects on either outcomes (HR = 1.00).

© 2022 Park JJH et al. *JAMA Network Open.*

eFigure 4. Total Cumulative Trial Duration Comparisons Between a Platform Trial Versus Conventional Trials

a) Base case

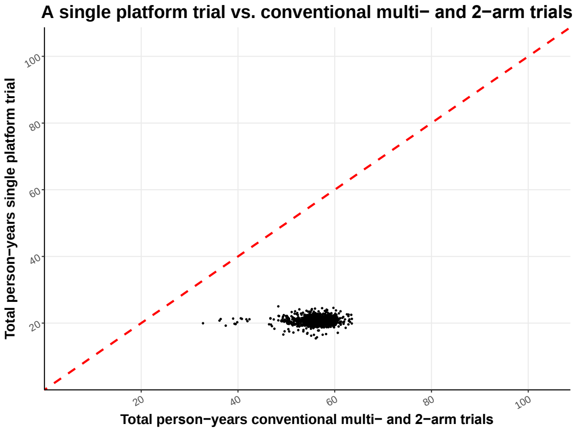

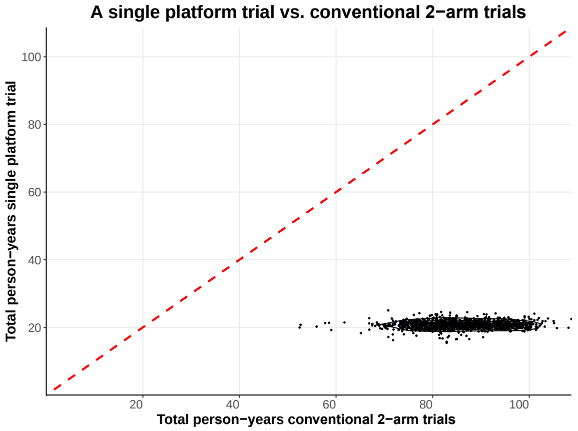

b) Worst case

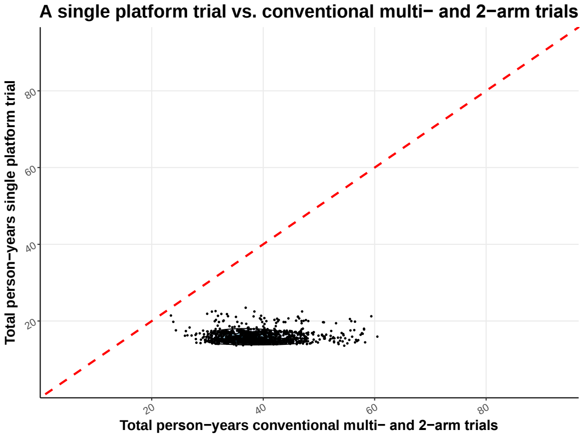

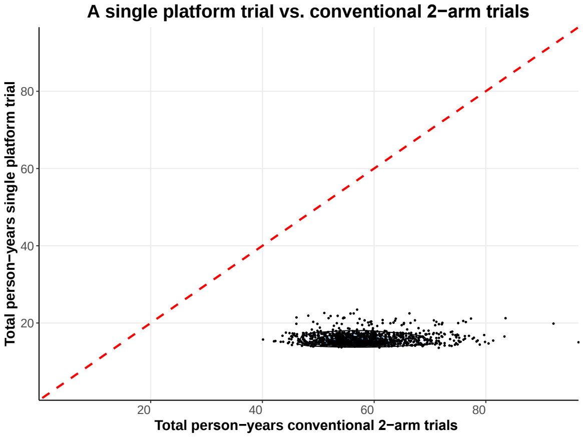

c) Best case

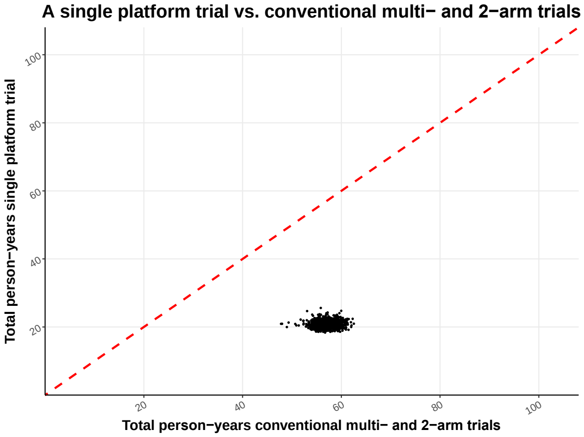

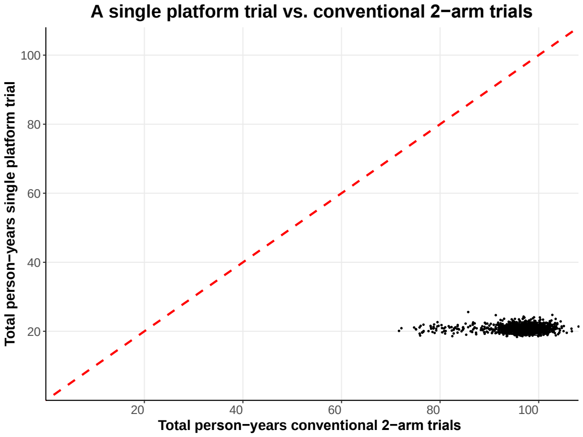

For interventions with reported treatment effects (hazard ratio [HR] and 95% confidence intervals) on FFS and OS, we used the point estimate as the ‘base case’, the lower confidence interval as the ‘best case’, and the upper confidence interval as the ‘pessimistic case’ for our simulations. For the last three intervention arms without reported results, we assumed the base case FFS and OS to have HR of 0.75, which was the target treatment effects by the STAMPEDE

© 2022 Park JJH et al. *JAMA Network Open.*

investigators. The best case scenario for these intervention arms assumed a treatment effect of 0.5625, twice as large treatment effects as the target effects, for both FFS and OS, and the pessimistic case scenario assumed that these interventions would have no treatment effects on either outcomes (HR = 1.00).

© 2022 Park JJH et al. *JAMA Network Open.*

eTable 4. Estimated Sample Sizes Between Different Clinical Design Scenarios

Estimated sample size	Scenario 1: A platform trial Mean (SD)	Scenario 2: A multi-arm + two-arm trials Mean (SD)	Scenario 3: Two-arm trials Mean (SD)	Scenario 2 vs 1		Scenario 3 vs 1	
				Mean difference (SD)	% difference Median (IQR)	Mean difference (SD)	% difference Median (IQR)
**Base case**							
No. of total patients randomized to treatments 1-10	4018.26 (193.659)	3783.621 (149.874)	4258.903 (242.555)	-234.639 (240.732)	-6% (-9.4%; -2%)	240.643 (319.232)	6.9% (1.2%; 11.4%)
No. of patients randomized to treatments 1-5	1811.181 (190.705)	1582.874 (137.346)	2208.64 (70.526)	-228.307 (230.855)	-13% (-19.8%; -4.7%)	397.459 (204.826)	20.8% (14%; 30.6%)
No. of patients randomized to treatments 6-10	2207.079 (27.005)	2208.64 (70.526)	2208.64 (70.526)	1.561 (76.184)	0% (0%; 0%)	1.561 (76.184)	0% (0%; 0%)
No. of total control patients for evaluation of all treatments (1-10)	2574.461 (81.019)	2949.383 (86.377)	4252.62 (237.345)	374.922 (118.257)	14.8% (12%; 17.7%)	1678.159 (248.058)	67.8% (58.3%; 72.7%)
No. of concurrent control patients for treatment 1-5	881.288 (47.175)	753.775 (63.997)	2203.375 (71.774)	-127.513 (80.255)	-14.6% (-19.2%; -9.6%)	1322.087 (86.811)	150.3% (141.2%; 160.6%)
No. of concurrent control patients for treatment 6-10	1693.173 (60.097)	2203.375 (71.774)	2203.375 (71.774)	510.202 (94.898)	30.5% (27.5%; 33.6%)	510.202 (94.898)	30.5% (27.5%; 33.6%)
**Pessimistic case**							
No. of total patients randomized to treatments 1-10	3519.609 (227.11)	3205.556 (312.262)	3619.2 (353.839)	-314.053 (399.004)	-9% (-16.1%; -0.8%)	99.591 (428.472)	2.9% (-5.8%; 10.9%)
No. of patients randomized to treatments 1-5	1444.448 (209.455)	1303.194 (178.23)	1751.83 (422.971)	-141.254 (282.929)	-9.4% (-20.5%; 2.5%)	307.382 (481.275)	23.9% (-9%; 50.3%)
No. of patients randomized to treatments 6-10	2075.161 (82.926)	1751.83 (422.971)	1751.83 (422.971)	-323.331 (433.558)	-9% (-35.7%; 4.9%)	-323.331 (433.558)	-9% (-35.7%; 4.9%)
No. of total patients randomized to control	2545.851 (104.65)	2694.985 (307.17)	3622.611 (347.691)	149.134 (324.763)	5.9% (-3.3%; 16.7%)	1076.76 (359.154)	42.7% (30.9%; 51.5%)
No. of concurrent control patients for treatment 1-5	888.551 (87.237)	791.654 (169.9)	1755.775 (415.109)	-96.897 (188.617)	-7.4% (-13.2%; -2.8%)	867.224 (426.602)	109.4% (48.4%; 142.7%)
No. of concurrent control patients for treatment 6-10	1657.3 (60.77)	1755.775 (415.109)	1755.775 (415.109)	98.475 (417.701)	13.5% (-19.1%; 31.5%)	98.475 (417.701)	13.5% (-19.1%; 31.5%)
**Best case**							
No. of total patients randomized to treatments 1-10	4216.884 (122.694)	3925.333 (95.94)	4406.308 (102.787)	-291.551 (153.757)	-7.2% (-9.1%; -4.8%)	189.424 (157.72)	4.4% (2.8%; 6.6%)
No. of patients randomized to treatments 1-5	2002.8 (122.089)	1711.381 (94.821)	2215 (0)	-291.419 (152.653)	-15% (-18.9%; -10.2%)	212.2 (122.089)	9.6% (6.3%; 14.4%)
No. of patients randomized to treatments 6-10	2214.084 (9.352)	2215 (0)	2215 (0)	0.916 (9.352)	0% (0%; 0%)	0.916 (9.352)	0% (0%; 0%)
No. of total patients randomized to control	2501.431 (84.65)	2921.378 (33.944)	4396.531 (101.475)	419.947 (91.981)	16.6% (14.1%; 19.5%)	1895.1 (133.15)	76.1% (72.4%; 80.1%)
No. of concurrent control patients for treatment 1-5	832.468 (39.93)	712.52 (30.892)	2210.03 (7.078)	-119.948 (51.398)	-14.3% (-17.8%; -10.8%)	1377.562 (40.8)	167.6% (158.5%; 174.5%)
No. of concurrent control patients for treatment 6-10	1668.963 (71.202)	2210.03 (7.078)	2210.03 (7.078)	541.067 (71.181)	31.9% (28.9%; 35.8%)	541.067 (71.181)	31.9% (28.9%; 35.8%)

© 2022 Park JJH et al. *JAMA Network Open.*

For interventions with reported treatment effects (hazard ratio [HR] and 95% confidence intervals) on FFS and OS, we used the point estimate as the ‘base case’, the lower confidence interval as the ‘best case’, and the upper confidence interval as the ‘pessimistic case’ for our simulations. For the three intervention arms without reported results, we assumed the base case FFS and OS to have HR of 0.75, which was the target treatment effects by the STAMPEDE investigators. The best case scenario for these intervention arms assumed a treatment effect of 0.5625, twice as large treatment effects as the target effects, for both FFS and OS, and the pessimistic case scenario assumed that these interventions would have no treatment effects on either outcomes (HR = 1.00).

© 2022 Park JJH et al. *JAMA Network Open.*

eTable 5. Total Cumulative Patient Follow-Up Time Between Different Clinical Design Scenarios

Simulated follow-up time (months): Mean (SD)	Scenario 1: A platform trial Mean (SD)	Scenario 2: A multi-arm + two-arm trials Mean (SD)	Scenario 3: Two-arm trials Mean (SD)	Scenario 2 vs 1		Scenario 3 vs 1	
				Mean difference (SD)	% difference Median (IQR)	Mean difference (SD)	% difference Median (IQR)
**Base case**							
Total follow-up for patients randomized to treatments 1-5 (months)	43958.745 (6650.624)	44160.714 (7515.323)	116314.288 (11223.959)	201.969 (9896.895)	1.4% (-13.9%; 17%)	72355.543 (13119.187)	168.3% (141.8%; 196%)
Total follow-up for patients randomized to treatments 6-10 (months)	82192.52 (5195.373)	116314.288 (11223.959)	116314.288 (11223.959)	34121.768 (12233.675)	42.1% (35.2%; 48.8%)	34121.768 (12233.675)	42.1% (35.2%; 48.8%)
Total follow-up for concurrent control for treatments 1-5 (months)	21820.179 (1908.674)	20833.18 (1669.713)	102920.136 (10226.48)	-986.999 (2643.762)	-3.6% (-10.8%; 3.3%)	81099.957 (10366.77)	377.9% (343.5%; 413%)
Total follow-up for concurrent control for treatments 6-10 (months)	101095.08 (6621.422)	102920.136 (10226.48)	102920.136 (10226.48)	1825.056 (12130.723)	2% (-3.5%; 7.9%)	1825.056 (12130.723)	2% (-3.5%; 7.9%)
**Pessimistic case**							
Total follow-up for patients randomized to treatments 1-5 (months)	31072.933 (6450.291)	28887.839 (6600.099)	39668.452 (25703.098)	-2185.094 (9449.75)	-8.7% (-22.9%; 13%)	8595.52 (26725.613)	5.2% (-44.8%; 90.8%)
Total follow-up for patients randomized to treatments 6-10 (months)	45009.443 (8147.032)	39668.452 (25703.098)	39668.452 (25703.098)	-5340.991 (26945.978)	-28% (-60.3%; 33.2%)	-5340.991 (26945.978)	-28% (-60.3%; 33.2%)
Total follow-up for concurrent control for treatments 1-5 (months)	23522.288 (3685.128)	19181.627 (4434.207)	39091.305 (24813.567)	-4340.66 (5690.323)	-16.4% (-22.6%; -9.6%)	15569.017 (25151.502)	38% (-26.1%; 150.1%)
Total follow-up for concurrent control for treatments 6-10 (months)	56866.417 (10877.751)	39091.305 (24813.567)	39091.305 (24813.567)	-17775.112 (26901.962)	-43.9% (-68.5%; 2.4%)	-17775.112 (26901.962)	-43.9% (-68.5%; 2.4%)
**Best case**							
Total follow-up for patients randomized to treatments 1-5 (months)	53979.925 (5579.283)	55275.371 (5662.129)	129366.694 (6516.321)	1295.446 (7960.05)	2% (-6.4%; 12.1%)	75386.769 (8501.664)	138.1% (121.7%; 156.7%)
Total follow-up for patients randomized to treatments 6-10 (months)	88328.665 (3909.603)	129366.694 (6516.321)	129366.694 (6516.321)	41038.029 (7424.752)	46.5% (40.5%; 52.9%)	41038.029 (7424.752)	46.5% (40.5%; 52.9%)
Total follow-up for concurrent control for treatments 1-5 (months)	20941.786 (1377.681)	20987.248 (1163.242)	103868.517 (5956.312)	45.462 (1769.345)	0.4% (-4.9%; 6.3%)	82926.731 (6123.275)	397.4% (369.5%; 425.5%)
Total follow-up for concurrent control for treatments 6-10 (months)	103905.838 (4162.234)	103868.517 (5956.312)	103868.517 (5956.312)	-37.321 (7270.004)	-0.4% (-4.5%; 4.8%)	-37.321 (7270.004)	-0.4% (-4.5%; 4.8%)

© 2022 Park JJH et al. *JAMA Network Open.*

For interventions with reported treatment effects (hazard ratio [HR] and 95% confidence intervals) on FFS and OS, we used the point estimate as the ‘base case’, the lower confidence interval as the ‘best case’, and the upper confidence interval as the ‘pessimistic case’ for our simulations. For the three intervention arms without reported results, we assumed the base case FFS and OS to have HR of 0.75, which was the target treatment effects by the STAMPEDE investigators. The best case scenario for these intervention arms assumed a treatment effect of 0.5625, twice as large treatment effects as the target effects, for both FFS and OS, and the pessimistic case scenario assumed that these interventions would have no treatment effects on either outcomes (HR = 1.00).

© 2022 Park JJH et al. *JAMA Network Open.*

eReferences:

1. Sydes MR, Parmar MKB, Mason MD, et al. Flexible trial design in practice - stopping arms for lack-of-benefit and adding research arms mid-trial in STAMPEDE: a multi-arm multi-stage randomized controlled trial. *Trials* 2012; **13**: 168.
2. James ND, Spears MR, Clarke NW, et al. Failure-Free Survival and Radiotherapy in Patients With Newly Diagnosed Nonmetastatic Prostate Cancer: Data From Patients in the Control Arm of the STAMPEDE Trial. *JAMA Oncol* 2016; **2**(3): 348-57.
3. Mason MD, Clarke NW, James ND, et al. Adding celecoxib with or without zoledronic acid for hormone-naïve prostate cancer: long-term survival results from an adaptive, multiarm, multistage, platform, randomized controlled trial. *Journal of clinical oncology* 2017; **35**(14): 1530.
4. James ND, de Bono JS, Spears MR, et al. Abiraterone for Prostate Cancer Not Previously Treated with Hormone Therapy. *N Engl J Med* 2017; **377**(4): 338-51.
5. Parker CC, James ND, Brawley CD, et al. Radiotherapy to the primary tumour for newly diagnosed, metastatic prostate cancer (STAMPEDE): a randomised controlled phase 3 trial. *Lancet* 2018; **392**(10162): 2353-66.
6. Hozo SP, Djulbegovic B, Hozo I. Estimating the mean and variance from the median, range, and the size of a sample. *BMC Med Res Methodol* 2005; **5**: 13.

© 2022 Park JJH et al. *JAMA Network Open.*
